# A novel approach to the quantitative analysis of the particulate matter in conventional cigarette smoke and heated tobacco product aerosols

**DOI:** 10.1016/j.heliyon.2024.e35028

**Published:** 2024-07-25

**Authors:** E.G. Tane, L. Martínez-Gómez, A. Amorós-Pérez, M.C. Román-Martínez, M.A. Lillo-Ródenas

**Affiliations:** Grupo MCMA, Departamento de Química Inorgánica e Instituto Universitario de Materiales de Alicante (IUMA), Facultad de Ciencias, Universidad de Alicante, Alicante, E-03080, Spain

**Keywords:** Heat-not-burn products, Heated tobacco products, IQOS, Combustible cigarettes, HCI, Quantitative analysis, Collection, Particulate matter

## Abstract

The particulate and soluble matter present in aerosols from combustible cigarettes (CCs) and Heated Tobacco Products (HTPs) was collected in liquid water. These liquids, yellowish in the experiments with cigarettes and colourless after using HTPs, were analysed by Laser Diffraction (LD) and by Transmission Electron Microscopy coupled to Energy Dispersive X-ray spectroscopy (TEM-EDX) to study the amount, size, composition, and other features of the particulate matter (PM) present in the collected aerosols.

The particulate matter concentration in HTPs samples is below the limit of quantification for LD, and only samples from cigarettes show a particulate matter concentration above such limit. TEM analysis has revealed that the liquid samples (from both, cigarettes and HTPs experiments) contain particulate matter, mainly composed of carbon (C) and oxygen (O), but also of traces of inorganic elements. The TEM electron beam results in the evaporation of the particulate matter derived from HTPs, but not of that derived from cigarettes, highlighting the different nature of the particulate matter in both systems, i.e. liquid particulate matter present in the HTPs aerosols and solid particulate matter in the cigarettes smoke. A protocol for the quantitative comparison of the particulate matter present in aerosols has been applied over sixteen TEM images for each sample, confirming important differences from the point of view of the amount of particulate matter and particle size ranges. Thus, the amount of particulate matter for HTPs aerosol samples is more than one order of magnitude lower than for cigarettes smoke.

## Highlights

• Analysis of the particulate matter generated in the use of tobacco products.
• Differences in physical and chemical characteristics, including size and volatility.
• Identification of the individual particles in the PM over 16 TEM images per sample.
• A statistical method has been designed to quantify the particulate matter by TEM.
• One order of magnitude difference in the PM in cigarette smoke and HTP aerosol.

**Table:** Abbreviations

A_P_	Area Occupied by Particulate Matter
A_T_	Total Area
CC	Combustible Cigarette
EDX	Energy Dispersive X-ray Spectroscopy
GC	Gas Chromatography
HCI	Health Canada Intense regime
HNBP	Heat-Not-Burn Product
HTP	Heated Tobacco Product
LD	Laser Diffraction
LF	Loading Factor
O_F_	Occupation Factor
PM	Particulate Matter
PM2.5	Particulate Matter with a maximum diameter of 2.5 μm
q × 3	Particle Size Distribution
Q × 3	Cumulative Size Distribution
QA1	Quantitative Analysis 1
QA2	Quantitative Analysis 2
QA3	Quantitative Analysis 3
TEM	Transmission Electron Microscopy
TOC	Total Organic Carbon

## 1 Introduction

Cigarette smoke has shown to be the cause of several health problems linked to the many and high levels of toxicants present in smoke as well as the solid fraction present in its particulate matter [1,2]. Solid particulate matter emissions are also recognized to be amongst the most critical environmental risks to public health [[3], [4], [5]]. In general, cigarettes smoke particulate matter is typically sub-micrometric and easily deposited in the human respiratory tract during smoking [2].

In recent years, new tobacco products have been developed as alternatives to traditional combustible cigarettes, including the so-called *heat-not-burn products* (HNBPs, also called HTPs, *heated tobacco products*) [1].

HTPs are based on heating tobacco at much lower temperatures than in combustible cigarettes (typically 250-350 °C versus 700-950 °C) [[6], [7], [8]] with the intention to significantly reduce the emission of toxicants, which are typically formed via different physico-chemical routes at the high temperatures achieved when tobacco combusts [4,9]. The combustion of tobacco releases volatile carbon-containing materials that partially form liquid-like particulate matter due to vapour condensation (homogeneous nucleation) and condensation of vapours on solid entities (heterogeneous nucleation) and can, as well, generate soot particles as a result of incomplete combustion [10]. In contrast, the use of HTPs involves vaporization and low temperature thermal decomposition of organic compounds present in tobacco, and the formation of new species by pyrogenesis and pyrosynthesis, but combustion-derived compounds and soot particles are not generated [3,6,9]. The aerosols emitted from HTPs are formed via homogeneous nucleation of the aerosol former glycerol, that is added to the tobacco substrates during processing, and evaporated when the tobacco substrates are heated [7,9]. The homogeneous nucleation of the vaporized glycerol upon cooling down results in an aerosol consisting of liquid-based particulate matter. Due to the low operating temperatures, the significant reduction in toxicants and the absence of soot particles, these products are claimed to have the potential to be low-risk alternatives to combustible tobacco products, such as cigarettes [7,10,11].

Despite its worldwide commercialization, there are very few studies focused on the detailed characterization and/or quantification of the particulate matter present in the aerosols generated by the HTPs and on the comparison with the particulate matter in smoke generated when smoking cigarettes. Thus [2,[10], [11], [12], [13], [14], [15], [16]], have paid attention to the sizes of the particulate matter in the mainstream smoke from combustible cigarettes and in mainstream aerosols from HTPs, in some cases also aiming to evaluate the composition of the particulate matter. Although some qualitative particulate matter characterization from cigarettes and HTPs (Amber box *HEETS* with *IQOS 3 MULTI*) was recently published by our research group [14,16], it was not possible to perform a quantitative comparison between the particulate matter emitted by cigarettes and HTPs, and even between different HTPs. In those two previous studies, we paid attention to two different particulate matter size ranges, analysed by laser diffraction, LD (particles of size ranging from 20 nm), and by transmission electron microscopy, TEM (particles of size ranging from 1 nm). Although in those studies the use of LD provided some quantitative information, in most cases the levels of the particulate matter were below the limit of quantification. In the case of TEM, in the previous studies the presence of PM was confirmed in a randomly selected area (or in some limited areas) of the TEM grids, and it could be confirmed if there was some PM present and, if so, give an estimation of the mean particle size. However, it was not possible to perform a fully representative quantitative comparison between the particulate matter emitted by cigarettes and HTPs, and even between different HTPs.

There exists a strong association between exposure to particulate matter with an aerodynamic diameter smaller than 10 μm and respiratory, cardiovascular, and neurodegenerative diseases or cancer [2]. However, it should be highlighted that not only the size of the PM, but also other physicochemical properties, such as its composition, are linked with their harmful effects [6] and, in this sense, recent studies have focused on comparing the PM from conventional cigarettes and HTPs from different perspectives [[17], [18], [19], [20], [21]].

Thus, to be able to assess the toxicological impacts of tobacco products when used, it is important to be able to precisely determine the size and composition of the particulate matter, as well as to elucidate whether combustion particles are being generated or not, and to evaluate different chemicals present in the aerosols. These are aspects that are often complex, as shown in the literature [[17], [18], [19], [20], [21]].

In a recent review, data on the concentration and size of particles, the total amount of particulate matter, and the composition of such matter detected in mainstream cigarette smoke or aerosol from HTPs are compiled and compared [20]. However, as the literature states, such comparison is not easy to carry out due to several reasons, including that: for each study the experimental conditions are very different and are not even clearly explained (making the comparison difficult); the puffing protocols are different between studies; blank experiments are omitted in many studies; the analytical methods used are not always suitable; and/or some studies omit to include a suitable number of replicates to ensure robust conclusions.

The present study complements and extends the published ones, focusing on the quantitative determination and characterization of particulate matter emitted from combustible cigarettes when smoked, and from HTPs during use. Thus, on the one hand, the present study extends the approach of particulate matter collection and analysis to additional devices and different heating technologies. On the other hand, the comparison of the particulate matter collected from the mainstream emissions after using tobacco products, both cigarettes and the different HTPs and their respective tobacco heating devices, is performed, also comparing them with blank tests. This is based on a protocol designed to systematically obtain and analyse the information from a large number of TEM images taken in fixed portions of the TEM grid for each sample. Also, another novelty of the present work is a more accurate estimation of the mean particle sizes, paying attention to the characteristics of individual particles constituting the detected particulate matter fraction.

To develop the protocol to systematically analyse particulate matter emitted by regular combustible cigarettes (Marlboro Red, Philip Morris USA Inc., Richmond, VA) and several heatsticks (HTP consumables) in combination with different heating devices, in particular, *HEETS Amber* variant heatsticks used with the *IQOS 3 MULTI* device (both bought in a local tobacco shop in Alicante); *HEETS Amber* variant sticks, used with *IQOS 3 DUO* (supplied by Philip Morris Products S.A., Neuchâtel, Switzerland) and *TEREA Regular* variant heatsticks used with *IQOS ILUMA* (supplied by Philip Morris Products S.A., Neuchâtel, Switzerland).

The *IQOS 3* (*MULTI* and *DUO*) and *IQOS ILUMA* devices have different heating technologies: *IQOS 3 MULTI* and *IQOS 3 DUO* include a resistive heating blade over which the tobacco product (heatsticks commercialized as *HEETS*) is inserted, while *IQOS ILUMA* operates using induction heating via an induction coil in the device and a metal insert in the heatstick (commercialized as *TEREA*) (see Figure SM1 in the Supplementary Material).

Once this new systematic approach has been presented, it is important to highlight that it is based on well-established analytical techniques, which allow quantifying the particulate matter content. For example, to analyse the PM collected in the liquid phase, such as in the present study, laser diffraction is a very interesting tool widely used for quantitative purposes. However, previous studies [14,16] have shown that the particulate matter collected after the use of some tobacco products can be below the optimum limit of quantification. This makes it necessary to use other techniques and systematic methodologies for the quantification of particulate matter, such as the approach developed and described in this paper.

## 2 Materials and methods

### 2.1 Tobacco products

All the tobacco products used in this study (cigarettes and heatsticks) were produced by Philip Morris International.

The cigarettes used were Marlboro Red. The different heatsticks and devices employed for their use are collected in Table 1, together with the nomenclature used in this research.

**Table 1:** Nomenclature of the heatsticks and devices analysed in the research.

Nomenclature	Heatstick	Device
HTP1	*HEETS Amber*	*IQOS 3 MULTI*
HTP2	*HEETS Amber*	*IQOS 3 DUO*
HTP3	*TEREA Regular*	*IQOS ILUMA*

### 2.2 Experimental setup and experiments performed

The experimental laboratory setup used (Figure SM2 in the Supplementary Material) is the same as in previous works [14,16], and was designed to trap any particulate matter and soluble compounds from mainstream emissions from tobacco products in liquid media (i.e., in 250 mL of distilled water), whose characterization and quantification is the objective of the present research. The cigarettes smoke or HTPs aerosol is forced to flow through the water contained in a gas washing flask with the aid of a vacuum pump. To increase the contact time of the smoke/aerosol with water, a holey rubber piece (0.6 mm holes) is placed at the end of the glass tube, which drives the smoke/aerosol and produces small bubbles.

In the setup, the flow rate was optimized and adjusted to a value consistent with realistic puffing experiments. This flow rate was determined by measuring, during a fixed time, the volume of water displaced in a graduated cylinder (see Figure SM3 in the Supplementary Material). Using a reduced pressure of 350 mbar (and measuring the flow rate before each experiment) the flow rate was approximately 1200 mL/min (note that in realistic puffing experiments the flow rate ranges between 500 and 1500 mL/min [22]). These experimental conditions were selected to obtain puffs in accordance to the Health Canada Intense (HCI) puffing regime (55 mL puff during 2 s every 30 s). Puffing was simulated with the help of a glass stopcock, and experiments were carried out using either 15 cigarettes or 15 heatsticks to get representative samples for each tobacco product type tested.

To collect particulate matter from conventional cigarettes and HTPs, Elix10 pure water was used (resistivity>5MΩ·cm at 25 °C, normally the range is 10–15MΩ·cm, and the total organic carbon (TOC) content < 30 ppb).

The following blank and puffing experiments have been performed:

1 Blank experiments:
  a Without tobacco products placed in the setup, to collect any potential dust present in the air flow and to determine any impurity present in the distilled water (refered to as Blank A, or water Blank A)
  b With the unlit/non-heated tobacco-products placed in the setup, to collect any small tobacco fragments that could be dragged by the air flow. This blank experiment is done for each of the four tobacco products, i.e. cigarettes, HTP1, HTP2 and HTP3 (Blanks B).
2 Puffing experiments:

Carried out with the different tobacco products (lit cigarettes and heated HTPs with different devices (HTP1, HTP2, and HTP3, in Table 1)).

### 2.3 Analysis techniques

The particulate matter collected in water after the completion of the different blank and puffing experiments was analysed using the following techniques:

**Transmission Electron Microscopy (TEM)** coupled to **energy dispersive** **X-ray** **spectroscopy (EDX)**. TEM images give information on the shape, mean size, and size distribution of the nanometric observed particulate matter, while EDX provides information about the elemental composition of these particulate matter. Two TEM equipments have been used: (a) TALOS F200x operating at 200 kV and (b) JEM-1400 Plus operating at 120 kV. In general, the magnification used in the measurements allows to characterize particulate matter of size ranging from 1 to 10 000 nm. For each microscopy analysis, one drop of liquid from the gas washing flask, representative of the solution, is poured on a 3.05 mm diameter TEM grid (based on Cr, Fe, Cu, Os and C) and dried after evaporation. For the analysis of the particulate matter content and for the particle size distributions, two different drops from this total volume were poured on two different TEM grids (one drop each) for comparison. In the present study, both bright field and dark field transmission electron microscopy images have been obtained, and are compiled. The approach to quantify the particulate matter content and to assess the particle sizes was done using the bright field TEM images.

EDX analysis was carried out focusing the electron beam on the area where particulate matter was located, and checking that the contribution of the composition of the grid on the analysis was minimum. Two EDX analysis were done for each sample. Dark field TEM images have been obtained when performing the compositional EDX analysis, so as to remark the particles/zones where the EDX analysis was performed.

In the present study, the quantitative analysis of particulate matter observed by TEM was carried out with samples obtained from the blank and puffing experiments using all the investigated tobacco products.

For the quantitative analysis of the TEM images, two different protocols have been used.

Protocol A It has been established to perform a simple and “fast” comparison between the particulate matter collected in water from blank experiments and from puffing experiments. The analysis is done in images where a certain number of particles can be observed and using images obtained with the TALOS F200x TEM equipment, in which the particle sizes (the largest dimensions) were measured.

**Fig. 1:**
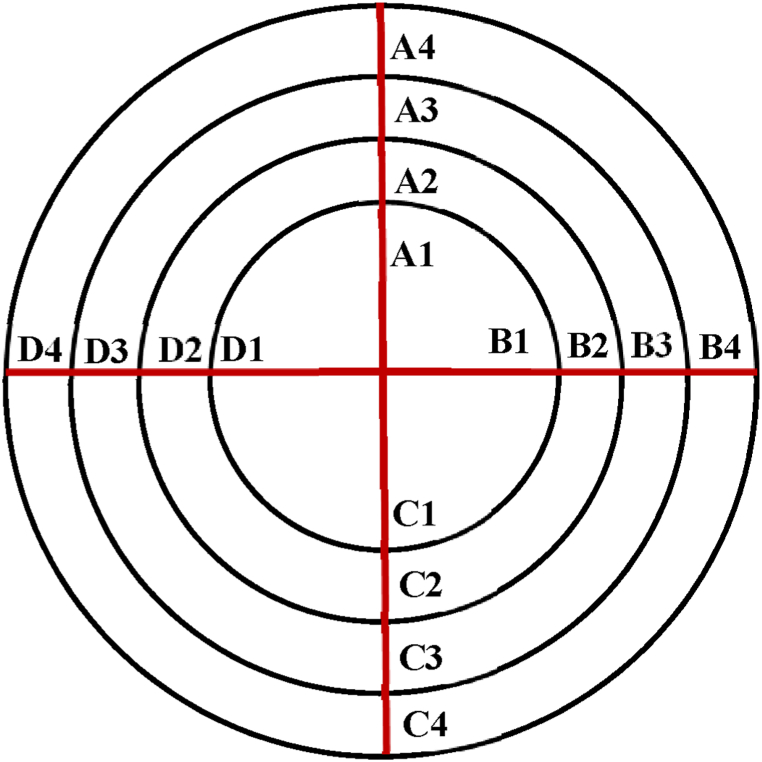
Location of the 16 areas (and the nomenclature given) of each TEM grid where TEM images at a magnification of 3000 were taken.

Protocol B This is a specific protocol to quantify particulate matter and to determine mean particle sizes and distributions. A fixed number of 20 TEM images were obtained for each liquid sample using the JEM-1400 Plus TEM microscope. These images include: one general view of the TEM grid at a magnification of 15, three images at magnification of 300 (one at the grid centre, another one in an area where many particles/aggregates were detected, and a third one in an area with no particulate matter), and sixteen images at a magnification of 3000 at selected positions of the grids. These sixteen images are taken at fixed positions of the TEM grid, locations schematically shown in Fig. 1 (from A1 to D4).

**Fig. 2:**
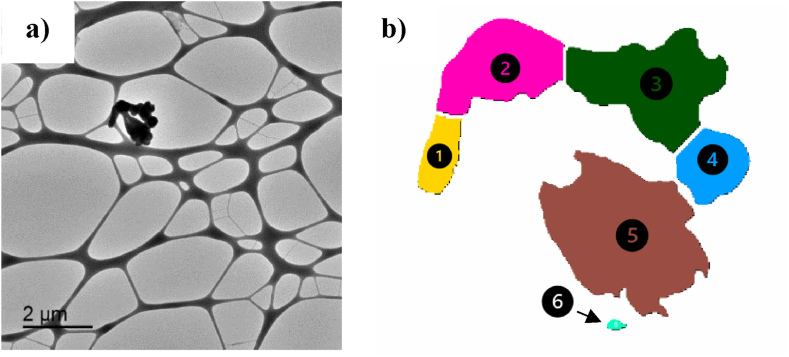
(a) Example of the TEM image of a sample from cigarettes experiment and (b) individual particles matter identified in the detected particulate matter in the image (a) using the ImageJ software.

The area "covered" by the detected particulate matter was determined using the software ImageJ. Individual particles constituting the detected particulate matter were identified first with the aid of the ImageJ software (based on contrast differences, see Fig. 2).

To determine the amount of particulate matter present in each image, an occupation factor (O_F_) has been calculated as the ratio of the total area occupied by particulate matter (A_P_) and the total area of each image (A_T_) (O_F_ = A_P_/A_T_·100, expressed in %).

For example, in Fig. 2, A_P_ is 92 200 nm^2^, A_T_ is 1.25·10^7^ nm^2^, and thus, O_F_ is 0.74 %.

In order to provide size values of the irregularly shaped particulate matter, its area is determined and, then, the diameter of a circle of that surface area is calculated, and chosen as the representative size of the particulate matter. Taking this into account, Table 2 shows as an example the diameters determined for the detected particles shown in Fig. 2.

**Table 2:** Area of the analysed particulate matter in Figure 2 and representative size (diameter of a circle of the same area).

Particle number	Area (nm^2^)	Representative size (nm)
1	17 490	149
2	23 880	174
3	5080	80
4	8400	103
5	37 050	217
6	300	20

The sample of Blank A has been used as a reference to normalize the particulate matter content. Then, the relative particulate matter content for a sample has been calculated as the ratio between the occupation percentage in that sample and the one in this blank.

**Scheme 1:**
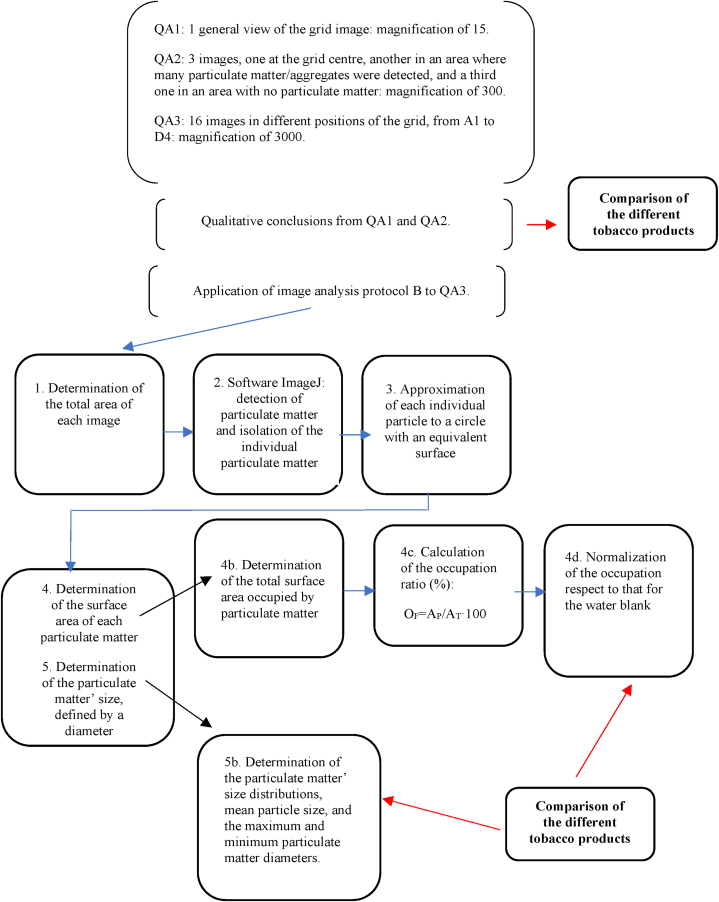
Detailed analysis scheme for the comparison and quantification of the collected particulate matter in the different samples.

The steps for this quantitative analysis are shown next on Scheme 1.

Based on Scheme 1, established to enable a quantitative comparison, the analysis protocol (Protocol B) was designed and 20 images in fixed portions of each TEM grid were obtained for each sample. All the TEM images obtained and used for the analysis with protocol B for Blank A and for all the puffing experiments are compiled in the Supplementary Material (Figure SM4 to SM11 for the Blank A; Figures SM12 to SM19 for the puffing of lit cigarettes sample; Figures SM20 to SM27 for the puffing of heated HTP1 sample; Figures SM28 to SM35 for the puffing of heated HTP2 sample, and Figures SM36 to SM43 for the puffing of heated HTP3 sample).

**Laser diffraction (LD)** allows the analysis of particulate matter using the Mie scattering theory (a mathematical-physical theory of the scattering of an electromagnetic plane wave by a homogeneous sphere) [23]. In the present study, LD has been used to evaluate the concentration and size distribution of the particulate matter suspended in the water samples collected in all the blank and puffing experiments. The equipment used was a SYNC analyser from MICROTRAC.

To obtain reliable results with this technique, an optimum loading factor (LF) of the solutions (related with the concentration/number of particulate matter) is necessary, refered to as the limit of quantification. In the equipment such a factor is 0.250, whereas the limit of detection is 0.055. Note that if 0.055 ≤ LF ≤ 0.250, the determined concentration of suspended particulate matter will be inaccurate, but information on mean particle sizes and distributions is still valid, as indicated by the equipment provider. The equipment has also provided, in some samples, particle size data for LF values below the theoretical limit of detection. In case so, these values have been reported in italics.

For the analysis of the particulate matter content and for the particle size distributions a volume of 160 mL (from the total 250 mL of each experiment) was analysed. Triplicate measurements of the same sample were performed, yielding the average values (mean size and loading factor).

**Gas chromatography (GC)** has been used to analyse the gases generated in the puffing experiments, using all the tobacco products studied. While it is well-established that cigarette smoking involves combustion and HTPs are designed to heat tobacco without combustion, determination of CO and CO_2_ concentration in the emissions was used to evaluate whether the tested HTPs operated as intended (without combustion) during the tests. The gas sample for analysis (250 μL) was extracted with a syringe from the downstream line to the glass stopcock (extraction was done in the position marked in Figure SM2) and injected in the gas chromatograph, Agilent 8860, equipped with a Thermal Conductivity Detector and two packed columns (Porapak-Q and MolSieve-13X)). The column temperature was 180 °C and He (17 mL/min) was used as carrier gas. For the analysis, the sample was manually injected in the inlet (heated at 225 °C) to the loop, where it was mixed with the carrier gas. Firstly, the mixture passed through the Porapak-Q column during 0.5 min for CO_2_ retention (1.1 min) and next, the gas passed through MolSieve-13X during 0.5 min for CO retention (2.7 min).

## 3 Results and discussion

### 3.1 Blank experiments

The water samples collected from blank tests (A (no tobacco products placed in the setup, to detect any potential dust in the air and/or any impurity in the distilled water) and B (for experiments with unlit/non-heated tobacco products)) is colourless, and no particulate matter is detected with the naked eye in any of them.

#### 3.1.1 TEM and EDX analysis

**Fig. 3:**
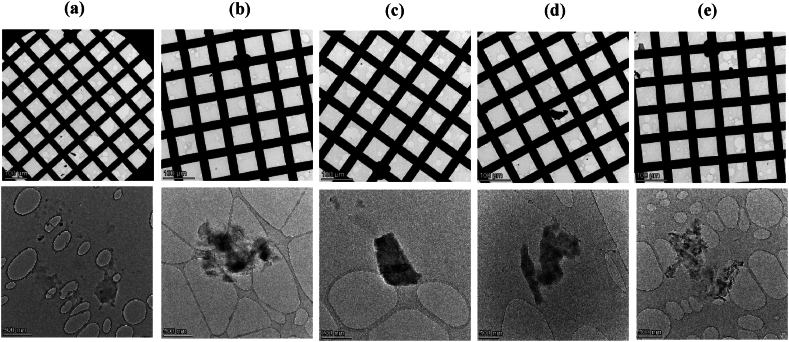
TEM images of water samples of blank experiments: (a) Blank A, for an experiment with no tobacco products placed in the setup; (b)–(e) Blanks B, experiments using unlit cigarettes and non-heated (b) HTP1, (c) HTP2 (d), and HTP3 (e). Images obtained with the TALOS F200X microscope.

Fig. 3 shows the general view of the grids (upper images), and the particular TEM images of the water samples collected in the blank experiments.

The general view of the grids shows that, in general, a very a low amount of particulate matter is observed. Regarding the particular images, for the sample of Blank A (Fig. 3a), particulate matter was scarcely found, while in the case of samples corresponding to Blanks B (Figures b–e), some particulate matter (small and without defined structure) can be observed.

**Fig. 4:**
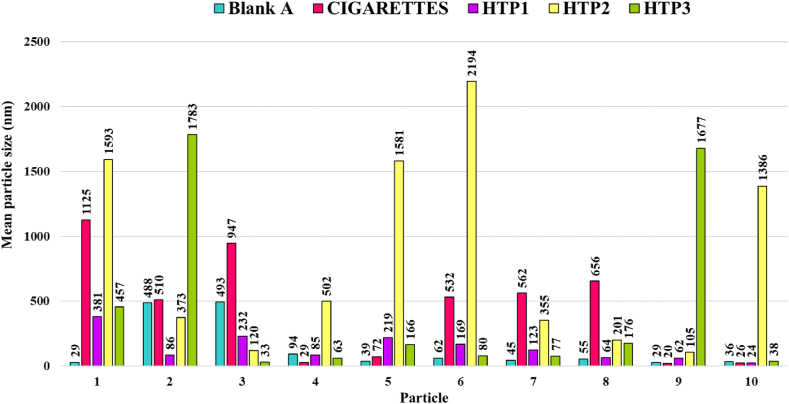
Sizes of 10 particles in water samples obtained in Blank A (carried out with no tobacco products placed in the setup) and in Blanks B (carried out with unlit cigarettes, or non-heated HTP1, HTP2 or HTP3). (Data obtained from TEM images, TALOS F200x, protocol A).

The particles in the TEM images from blank tests have been analysed following Protocol A (and focusing on 10 particles). A broad particle size distribution, in the range from 20 to 2200 nm, has been obtained (Fig. 4). For Blank A, particles of sizes up to about 500 nm have been detected. For Blanks B (the experiments with unlit cigarettes, and non-heated HTP1, HTP2 or HTP3), the particle size strongly depends on the tobacco product used. In general, they are larger for HTP2 and smaller for HTP1. These particles may be solid fragments carried by the air during puffs (like dust) and/or extracted from the tobacco product or the filter.

**Fig. 5:**
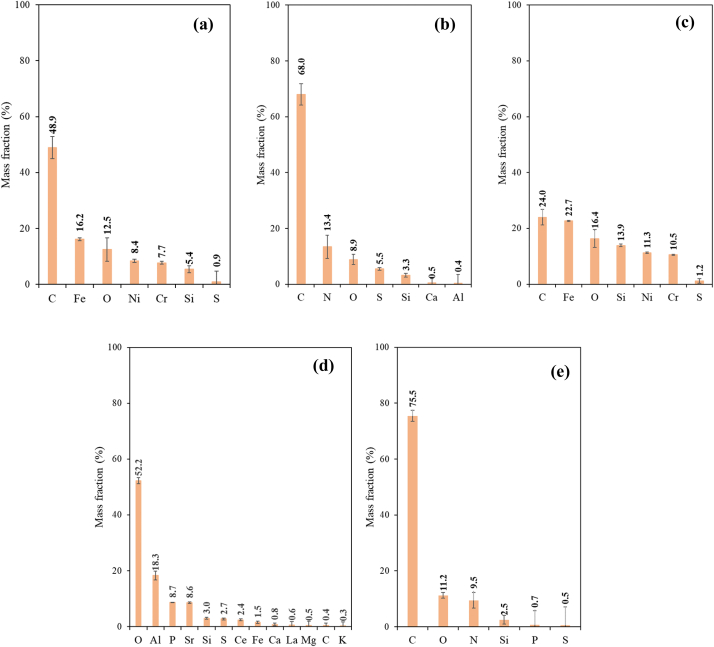
Mass fraction values of elements identified by EDX on blank experiments with TALOS F200X: (a) Blank A, (b)–(e) Blanks B with unlit cigarettes (b), and non-heated HTP1(c), HTP2, (d) and HTP3 (e).

Fig. 5 shows the mass fraction values of the elements identified by EDX in the particulate matter detected in the blank experiments. EDX analysis has revealed that the particulate matter is found in all these blank experiments, and it presents a variable composition, as expected considering that dust and/or tobacco derived fragments can be related with such matter. Together with carbon and oxygen, a large variety of metals are detected. It is important to highlight that in the Blank A analysis, some elements related to dust, such as Fe, Ni, Cr, Si and S, are found. These elements are also present in the Blanks B**,** with unlit cigarettes and non-heated HTP1, HTP2, and HTP3, together with others such as Ca, Al and other impurities that may come either from dust or from fragments of tobacco.

#### 3.1.2 Laser diffraction analysis

**Fig. 6:**
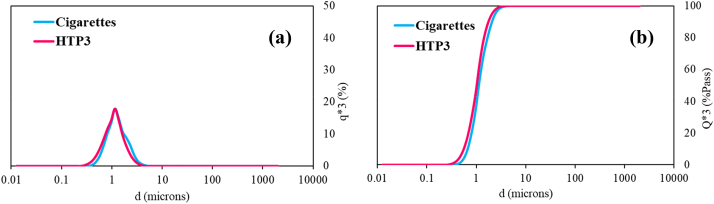
Laser diffraction data of samples of Blank B experiments carried out with unlit cigarettes and with non-heated HTP3: (a) Particle size distribution (q × 3) and (b) cumulative size distribution (Q × 3).

The LD analysis of the samples from Blank A did not show the presence of particulate matter. In the case of samples from Blanks B, only those corresponding to the experiments carried out with unlit CC and non-heated HTP3 show the presence of particulate matter (Fig. 6), with a mean size of ∼1.16 μm.

However, these data should be carefully considered. The loading factors in these blank experiments are very small, both below the limit of detection loading factor of 0.055. These low LF values are reasonable since a very low amount of particulate matter is expected in any of the blanks. Therefore, with this analysis, it cannot be confirmed that the detected particles are representative of the analysed samples.

### 3.2 Puffing experiments

**Fig. 7:**
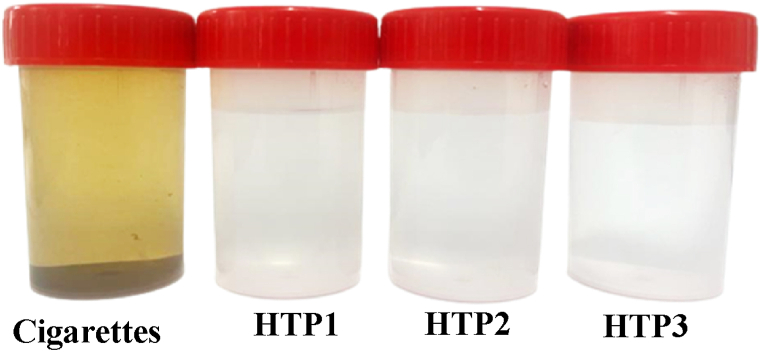
Photographs of collected liquid samples.

Fig. 7 shows images of the water solutions collected after testing with 15 lit cigarettes and 15 heated HTPs. The water samples collected from puffing experiments are yellowish in the case of cigarettes smoking, while they remained colourless and clear in the experiments carried out with HTPs. This reflects a larger amount of particulate matter in the case of cigarettes and could also reflect a different nature/composition. These results agree with those obtained by refs. [14,16].

#### 3.2.1 TEM and EDX analysis

##### 3.2.1.1 Protocol A

**Fig. 8:**
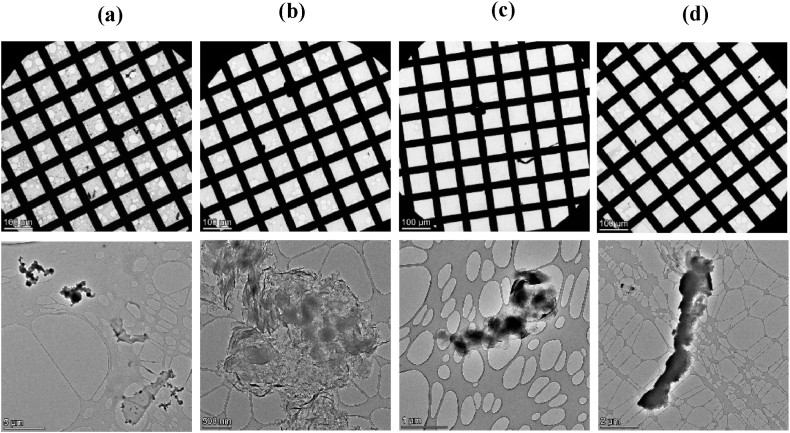
TEM images of water samples from puffing experiments with: (a) lit cigarettes and heated (b) HTP1, (c) HTP2 and (d) HTP3 (using TALOS F200x).

Fig. 8 shows a general view of the grids containing the different samples analysed, and particular TEM images for each sample.

Fig. 8a shows that in the samples collected in puffing experiments performed with lit cigarettes, a high population of solid particulate matter with well-defined homogeneous spherical structures was observed, in agreement with the results obtained by refs. [14,16]. Their characteristics are typical of solid particulate matter formed during combustion. However, other solid particulate matter may also be present, such as air dust and related inorganic particulate matter.

In contrast, Fig. 8b, 8c and 8d show that the deposited particulate matter from puffing experiments with heated HTPs has the appearance of droplet-like agglomerates, without a clear indication of primary particles. The particulate matter present in samples derived from HTPs was found to be beam sensitive and it was partially decomposed during the TEM imaging, in agreement with previous works [10]. This suggests that HTP-derived particulate matter is not a massive solid substance but more liquid phase-related one, in contrast to the particulate matter derived from cigarettes.

TEM images confirm that the amount of deposited matter from HTPs aerosols was much lower than the collected particulate matter from conventional cigarette smoke. After a thorough search over the TEM grid, some particulate matter was observed, showing an agglomerated structure with different shapes and droplet-like agglomerate appearance with a wide range of sizes (Fig. 8). This particulate matter seems to be mainly formed by condensates of glycerol and other compounds present in the generated aerosol. There is a stark contrast between the solid well-defined particles observed in the case of cigarettes, and the amorphous, undefined particles in the case of HTPs aerosols, highlighting the different nature of the particulate matter from cigarettes and HTPs.

**Fig. 9:**
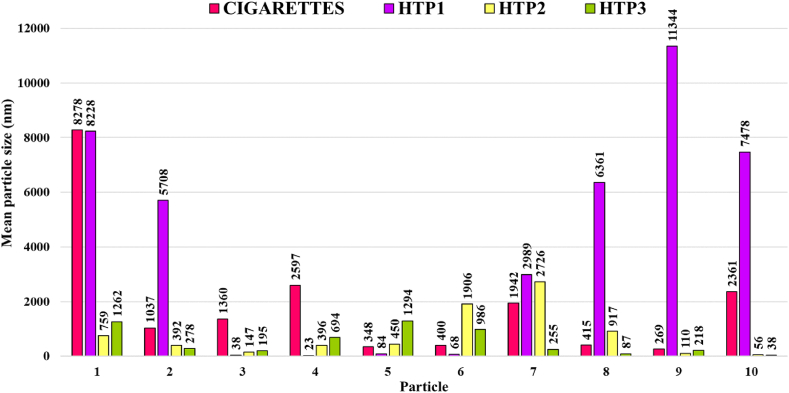
Sizes of 10 particles detected in samples from lit/heated puffing experiments (analysed by the Protocol A on TEM images using TALOS F200x).

Fig. 9 compiles the size distribution determined from the analysis of 10 particles observed in TEM images of any of the water samples collected in the puffing experiments carried out with the lit cigarettes and heated HTPs tested. The data displayed in Fig. 9 confirms that, for cigarettes and HTP1, the maximum particle sizes observed are larger in the puffing (lit/heated) experiments than in the corresponding (unlit/non-heated) Blanks B, while for HTP2 and HTP3, for the blanks and the puffing experiments they are in the same size range.

Table 3 summarises the main data from Fig. 9 (and from Blank A in Fig. 4, included for the sake of comparison).

**Table 3:** Particle size ranges (in nm, corresponding to the largest dimension) in samples obtained from blank experiments and puffing experiments.

Test	Particle sizes (nm) in blank experiments (Fig. 4)	Particle sizes (nm) in puffing experiments (Fig. 9)
Blank A	29 to 493	
Cigarettes	20 to 1125	270 to 8300
HTP1	24 to 381	23 to 11 344
HTP2	105 to 2194	56 to 2726
HTP3	33 to 1783	38 to 1294

For the analysis following the Protocol A in Fig. 9, and the comparison with Fig. 4 blanks, it can be stated that the sizes of the particles detected in the puffing experiments, ranging from 23 to 11 344 nm (see Table 3), are rather larger than the particles detected in blank samples, except for HTP2 and HTP3. This information, together with the qualitative observation of the difference in the amount of particulate matter, much larger in puffing experiments than in blank ones confirms, as expected, that most of the particulate matter in tobacco-related products is generated during their operation, which is being analysed next in detail.

##### 3.2.1.2 Protocol B

Considering the previous conclusions, a large effort has been dedicated to an accurate determination of the mean sizes and to the quantification of the content of the detected particulate matter in puffing experiments.

Focusing on the TEM data using a 15x magnification, in the general view of the grid images (see Supplementary Information), no significant differences were observed between HTPs samples (Figures SM20, SM28 and SM36 for HTP1, HTP2 and HTP3, respectively) and the water Blank A (Figure SM4), while for the cigarettes sample (Figure SM12), it is possible to distinguish aggregates of particles.

From the images taken at a magnification of 300x, some particulate matter was observed on the grid centre imaged of the cigarettes sample (Figure SM13). For HTP1 (Figure SM21), some particulate matter was observed at the grid centre, while for HTP2 and HTP3 (Figures SM29 and SM37, respectively), no particulate matter was observed at a magnification of 300x (no differences with the water Blank A centre grid, Figure SM5). Looking for areas where many particulate matter/aggregates were detected, a high population of spherical particles were observed for cigarettes samples (Figure SM14), while very few locations with particulate matter, and only after a thorough search, were detected for HTPs samples and for water Blank A (Figures SM6, SM22, SM30 and SM38 for water Blank A, HTP1, HTP2 and HTP3 samples, respectively). It was complicated to find an area with no particulate matter for cigarettes samples (notice that some particles were observed in Figures SM15), while for HTPs samples (Figures SM23, SM31 and SM39, for HTP1, HTP2 and HTP3, respectively) and for water Blank A (Figure SM7), most of the grid was absent of particulate matter.

Beyond the qualitative analysis, Protocol B aimed to perform a quantitative analysis using, for each sample, the information provided by 16 TEM images taken at 3000x magnification (see Supplementary Material). Thus, Table 4 compiles, for each sample, the total area occupied by particulate matter (A_P_) and the total area of each image (A_T_) and the percentage of area occupied by particulate matter (O_F_, expressed as %) and, also, the relative contents of particulate matter (normalized ascribing the value of 1 to the particulate matter content in water Blank A). This table highlights that the relative content of particulate matter is much larger in cigarettes samples, even more than an order of magnitude larger than HTPs samples. For the HTPs, HTP3 showed the highest occupation factor of the heated HTPs, followed by HTP2 and HTP1. These results evidence that the combustion of tobacco and the resulting high temperatures in cigarettes lead to much larger amount of particulate matter than when tobacco is heated instead of combusted in HTPs.

**Table 4:** Total area of each image (A_T_), total area occupied by particulate matter in the image (A_P_), percentage of area occupied by particulate matter (O_F_, expressed as %) and relative content of particulate matter in the samples from puffing experiments, normalized ascribing the value of 1 to the particulate matter content in the water Blank A, whose data are compiled in the first raw.

Sample	A_T_ (nm^2^)	A_P_ (nm^2^)	O_F_ %	Relative content of particulate matter
Blank A	1.99 10^8^	4104	0.02	1
Cigarettes	1.99 10^8^	1.90·10^7^	9.53	476
HTP1	1.99 10^8^	2.29·10^5^	0.12	6
HTP2	1.99 10^8^	4.02·10^5^	0.20	10
HTP3	1.99 10^8^	1.53·10^6^	0.77	38

Table 5 summarises the mean size (radius, in nm) and the smallest and largest particulate matter present in each sample (determined from the TEM images at a magnification of 3000 from the Supplementary Material) applying the Protocol B.

**Table 5:** Mean particulate matter size and size range (nm) for Blank A and the puffing experiments (lit cigarettes and heated HTP1, HTP2 and HTP3 samples).

Sample	Mean size (nm)	Particulate matter size range	
		Min size (nm)	Max size (nm)
Blank A	20	3	52
Cigarettes	62	1	750
HTP1	51	3	153
HTP2	18	2	190
HTP3	23	2	216

This table highlights that there are important differences between the sample from cigarettes and those from HTPs from the point of view of sizes. Thus, the maximum sizes for cigarettes are much larger than for any HTPs, and also larger than for the water Blank A. Also, the mean size is much larger for cigarettes-particulate matter, followed by HTP1, and then for HTP3 and HTP2, which, in fact, are in the same range as in the Blank A.

##### 3.2.1.3 EDX data

**Fig. 10:**
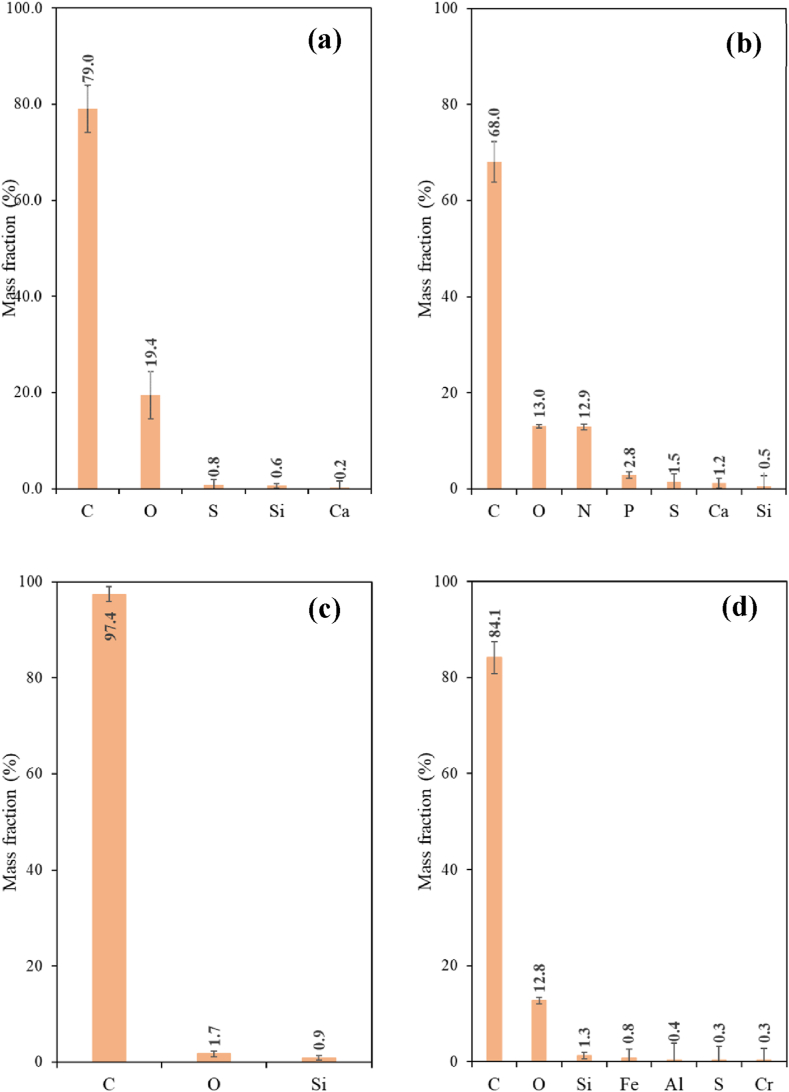
Mass fraction values of elements identified by EDX from puffing experiments with: (a) CC, (b) HTP1, (c) HTP2 and (d) HTP3 (using TALOS F200x).

The EDX analysis of selected particulate matter found in the water samples of the puffing experiments has been carried out with the TALOS F200x microscope. Fig. 10 includes the obtained results for the elemental composition of these particles (these data are obtained from two portions of each sample).

From data of Fig. 10 it can be concluded that in both CCs and HTPs samples, the detected particulate matter mainly contains carbon (C) and oxygen (O), in agreement with [10]. Other detected elements are: nitrogen (N), aluminium (Al), silicon (Si), phosphorus (P), sulphur (S), chloride (Cl), potassium (K), calcium (Ca), titanium (Ti) and iron (Fe). It should also be considered that some impurities could be present in the water originated from the tobacco and/or wrapping paper or carried from the flowing air. Such impurities could be responsible for the other compounds different from C and O present in, for example, the HTP1 sample (see Table 6).

**Table 6:** Semiquantitative EDX information, C, O and other elements (in wt. %) content in cigarettes and HTPs samples (for lit cigarettes and heated HTPs puffing experiments).

Sample	C (wt.%)	O (wt.%)	Other (wt.%)
Cigarettes	79	19	2
HTP1	68	13	19
HTP2	97	2	1
HTP3	84	13	3

Another experimental observation that should be highlighted is the sensitivity and easy evaporation during the TEM imaging of the particulate matter derived from HTPs, suggesting that the particulate matter in the HTPs aerosols is in liquid phase and verifies, in agreement with the results by ref. [10], showing the absence of solid organic particles in HTPs aerosol. The deposited HTPs aerosol particulate matter appeared to be featureless without individual particles visible. Such liquid particulate matter is likely formed from vaporized organic material (i.e. tobacco volatiles, glycerol and other additives), and is in agreement with the main detected elements (carbon (C) and oxygen (O) (Table 6). The observed inorganic elements present are: aluminium (Al), magnesium (Mg), silicon (Si), potassium (K), calcium (Ca), sodium (Na) and sulphur (S).

#### 3.2.2 Laser diffraction (LD)

**Fig. 11:**
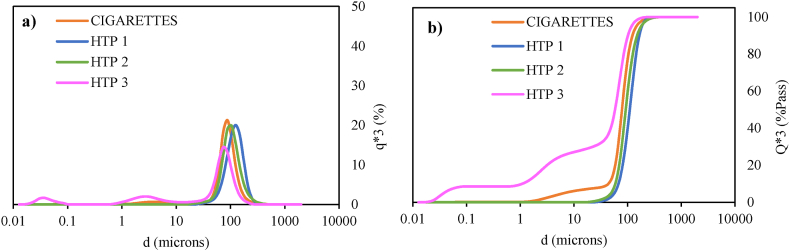
Size distribution of particulate matter (aggregates) in puffing experiments samples. (a) Particle size distribution (q × 3) and (b) cumulative size distribution (Q × 3).

Fig. 11 shows the size distribution of the particles detected by LD, and highlights that the mean sizes are in the same range for all the samples (75 –125 μm), with unimodal size distributions in the case of cigarettes, HTP1 and HTP2, and trimodal for HTP3.

Table 7 highlights that only for the cigarettes sample the loading factor is very close to the limit of quantification (LF > 0.250). This reflects that although similarities in mean sizes of the particle aggregates were found for cigarettes and HTPs, the particulate matter concentration is significantly higher in cigarettes sample compared to that in HTPs samples, which agrees with the TEM analysis. Specifically, for HTP3 sample, the LF is below 0.055, the limit of detection. Thus, particulate matter present in HTP3 solution can be considered negligible. Note that particulate matter is collected in water (and this liquid media is characterized by LD) and, as a result of that, there can be agglomeration and/or coalescence and the reported results of particles sizes, and shapes do not reflect the properties of the aerosolised particulate matter in the emitted cigarettes smoke or HTPs aerosols. This explains why these mean sizes are far larger than particulate matter in the breathable range (<PM2.5 μm).

**Table 7:** Mean size (nm) of the particulate matter (aggregates) and loading factor of cigarettes and HTPs puffing experiments samples using an analysis volume of 160 mL.

Sample	Mean size of aggregates (nm)	Loading factor
Cigarettes	8.80·10^4^	0.226
HTP1	1.24·10^5^	0.079
HTP2	1.05·10^5^	0.069
HTP3	*7.40·10*^*4*^	<0.055

#### 3.2.3 Gas chromatography

Aiming at comparing the difference in particulate matter resulting from cigarette smoking or HTPs usage from another point of view, the mainstream cigarettes smoke and HTPs aerosols generated were analysed by GC. CO and CO_2_ were analysed to give an indication of whether the tested products operated as intended, i.e. combustion of tobacco during cigarette smoking and only heating tobacco without combustion for the HTPs tested. In a first step, the air was analysed as blank, and the corresponding CO and CO_2_ values from the blanks were subtracted in the puffing experiments. The resulting percentages of CO and CO_2_ obtained are included in Table 8 for cigarettes and HTPs. It should also be recalled that blank experiments (unlit/non-heated ones) were also carried out, and the concentration of the CO and CO_2_ analysed gases are similar to those in blank air analysis.

**Table 8:** CO and CO_2_ (%) determined in the gas samples of the puffing experiments carried out with lit cigarettes and heated HTP1, HTP2 and HTP3 after subtracting the corresponding air blank data.

Sample	CO (%)·10^−2^	CO_2_ (%)·10^−2^
Cigarettes	28.8 ± 9.0	55.5 ± 19.6
HTP1	0.7 ± 1.0	23.6 ± 6.4
HTP2	0.0 ± 0.0	6.9 ± 1.4
HTP3	1.7 ± 0.1	36.0 ± 0.3

GC analysis of the gases generated in puffing experiments allows to conclude that the concentration of CO_2_ and, especially, CO in the gas phase of the cigarettes experiments is much higher than those found in the HTPs aerosols, which agrees with (Cozzani et al., 2020). Besides, water steam is detected in both cigarettes and HTPs. These findings help to confirm that no combustion occurs during the use of the tested HTPs and that they are operated as intended.

### 3.3 General discussion

All the liquids in the blank experiments remain colourless and clear. The TEM analysis of water samples from blank experiments shows that some well-defined carbon-based solid particulate matter, with a size range between 29 and 493 nm, is detected in the water when air flow passed through the system without any cigarettes or HTPs (Blank A), and the size range is between 20 and 2194 nm when unlit cigarettes or non-heated HTPs are placed in the experimental setup (Blanks B).

In general, the number/concentration of particulate matter only due to the air flow (Blank A) is very small and the same occurs in the Blanks B, obtained in experiments involving the air flowing through unlit cigarettes or non-heated HTPs.

Focusing on a quantitative approach, water samples from puffing experiments were analysed by LD and TEM. The corresponding sizes of the detected particulate matter by both techniques (using Protocol B) are summarized in Table 9.

**Table 9:** Mean sizes for the detected particulate matter analysed by LD, and mean and maximum sizes analysed by TEM (using Protocol B) for puffing experiments, for lit cigarettes and heated HTPs.

Sample	Mean size by LD (nm)	Mean size by TEM (nm)	Maximum size by TEM (nm)
Cigarettes	8.80·10^4^	62	750
HTP1	1.24·10^5^	51	153
HTP2	1.05·10^5^	18	190
HTP3	*7.40·10*^*4*^	23	216

Table 9 remarks the need of using complementary techniques to perform an accurate characterization of the particle sizes from cigarettes smoke and HTPs aerosols. For that reason, TEM and LD are suitable techniques, since they do not pay attention to the same particle size ranges. It should be recalled that in the present study, particulate matter is collected in water and measured in water by LD and, as a result of that, there is some agglomeration and/or coalescence of the particulate matter. This explains why these mean sizes are far larger than particulate matter in the breathable range (<PM2.5 μm).

Table 9 also remarks that the great difference in the maximum particle size by TEM for cigarettes sample respect to HTPs samples is another evidence of the different nature of the particulate matter in HTPs and CCs.

Hence, it should be recalled that not only the sizes of the particulate matter are important but also, and especially, the nature and composition of such particulate matter. Such information is of utmost importance to be able to carry out rigorous toxicological assessments of tobacco product emissions. As observed in the microscopy analyses of the deposited particulate matter from the HTPs aerosols before heating, the majority of the deposits consisted of organic carbonaceous particulate matter, producing undefined layers without observable individual solid particles on the sample grids. This suggests that the particulate matter emitted from HTPs is composed of liquid-based particles. In contrast, for CCs, well defined carbon-based solid particles (i.e. soot) originated from the combustion process were observed in TEM images. These results are compared with other reported works [10,11,14], where conventional cigarettes smoke was reported to contain a large number of carbon-based solid particles, while aerosols emitted from HTPs contain mostly carbonaceous liquid-based layers without a defined form. These findings support the outcomes of this study. From the LD analysis, the water sample from puffing experiments on smoked cigarettes contains particles with a mean particle size of 88 μm, while particles detected in water samples from collected HTPs aerosols can be considered negligible (LF < 0.250), in agreement with the results obtained in ref. [14]. On the other hand, it is important to highlight that the particulate matter analysed by LD is suspended in water, and it can form condensates or aggregates [14], whereas the particulate matter detected in TEM analyses results from an evaporated drop, and some fraction may correspond to water-soluble compounds that are isolated after the liquid evaporation. This can also explain the differences in the maximum sizes for particles from cigarettes and HTPs samples. It cannot be discarded that the evaporation of the more volatile fraction of the particulate matter takes place during TEM analysis, due to the used vacuum. This was especially observed for HTPs samples, where the liquid-based particulate matter evaporated under the TEM beam during the analysis.

All the results obtained results in this study for blank experiments, and for the puffing experiments with lit cigarettes and heated HTPs are summarized in Table 10, Table 11, respectively.

**Table 10:** Compiled TEM-EDX (TALOS F200x) and LD results for blank experiments (Blank A, with no tobacco products placed in the setup, and Blanks B, for unlit cigarettes and non-heated HTP1, HTP2 and HTP3).

Sample	TEM-EDX TALOS F200x				LD	
	Size range (nm)	C (%)	O (%)	Other (%)	Mean size (nm)	Loading Factor
Blank A	29–493	48.9	12.4	38.7	–	<0.055
Cigarettes	20–1125	68.0	8.8	23.2	*1.16·10*^*3*^	<0.055
HTP1	24–381	24.0	16.4	59.6	–	<0.055
HTP2	105–2194	0.4	52.2	47.4	–	<0.055
HTP3	33–1783	75.5	11.2	13.3	*1.16·10*^*3*^	<0.055

**Table 11:** Compiled TEM-EDX (TALOS F200x and JEM-1400) and LD results for puffing experiments with lit cigarettes and heated HTPs, and for Blank A (for comparative purposes).

Sample	TEM-EDX TALOS F200x				TEM-EDX JEM-1400			LD		GC	
	Size range (nm)	C (%)	O (%)	Other (%)	Mean size (nm)	Size range (nm)	O_F_ (%)	Mean size (nm)	LF	CO (%)·10^−2^	CO_2_ (%)·10^−2^
Blank A	29–493	48.9	12.4	38.7	20	3–52	1	–	–	–	–
Cigarettes	270–8300	79.0	19.4	1.6	62	1–750	476	8.80·10^4^	0.226	28.8 ± 9.0	55.5 ± 19.6
HTP1	23–11 344	68.0	13.0	19.0	51	3–153	6	1.24·10^5^	0.079	0.7 ± 1.0	23.6 ± 6.4
HTP2	56–2726	97.4	1.7	0.9	18	2–190	10	1.05·10^5^	0.069	0.0 ± 0.0	6.9 ± 1.4
HTP3	38–1294	84.1	12.8	3.1	23	2–216	38	*7.40·10*^*4*^	<0.055	1.7 ± 0.1	36.0 ± 0.3

## 4 Conclusions

The particulate matter present in the aerosol generated in puffing experiments carried out with combustible cigarettes and HTPs was analysed after being collected in water, in an experimental setup specifically designed for this purpose.

Blank experiments show the presence of a small amount of particulate matter in the collected water, corresponding to dust particulate matter from the environment air. However, the collecting water remained colourless and clear. From the puffing experiments, it can be concluded that the smoke generated from cigarettes led to a yellowish solution with dispersed, well-defined brown particulate matter, while the liquid solution remained colourless and clear in the experiments with HTPs.

TEM analysis of the water samples obtained from puffing tests with cigarettes reveals the presence in the smoke of a high population of solid particulate matter with well-defined homogeneous spherical structures. Their characteristics are typical of solid particulate matter formed during combustion. However, some dust present in the air may also appear in the detected particulate matter, such as inorganic particulate matter. The amount of deposited matter from HTPs aerosols is much lower than for cigarettes smoke, and it shows an agglomerated structure with different shapes without a clear indication of primary particulate matter. It was observed that the TEM electron beam resulted in the evaporation of the particulate matter derived from HTPs, but not of that derived from cigarettes, highlighting the different nature of the particulate matter originated in both systems, i.e. liquid particulate matter in HTPs aerosols and solid particulate matter present in cigarettes smoke.

The quantitative analysis of the amount and size of the observed particulate matter, performed for each sample over sixteen TEM images at fixed locations, shows that the relative particulate matter content (respect to blank data) for cigarette samples (476) was significantly higher than those for HTPs samples (from 6 to 38), and the size of the cigarettes particles presents a maximum of 750 nm, while for HTPs the maximum size for the particulate matter is 216 nm.

Regarding the laser diffraction characterization, only for cigarettes samples the loading factor was above the optimum quantification limit (LF > 0.250). The LF was far below the quantification limit for most of the HTPs. This confirms the much higher particulate matter concentration in cigarettes than in HTPs aerosol-derived samples.

GC analysis of the exhaust gases from cigarettes and HTPs confirmed that no combustion occurs during the use of HTPs, in contrast to what it is observed in the case of combustible cigarettes. This shows that the HTPs tested operated as intended, i.e. without combustion of the tobacco substrate.

The results of the characterization experiments carried out, both qualitative and quantitative, show a big difference in the amount and sizes, as well as the physical nature, of the particulate matter present in the smoke from combustible cigarettes in comparison with the particulate matter emitted from HTPs. These differences between the physical and chemical characteristics of the aerosols from HTPs compared to the smoke from cigarettes suggest that the toxicological profile is likely fundamentally different from combustible tobacco products and HTPs, heating tobacco instead of combusting it.

The method presented in this study is useful and relevant, not only for the characterization of particulate matter from tobacco sources, but in any field related with air and environment quality, especially when differences in particulate matter characteristics are studied.

## Funding

10.13039/100014729Philip Morris International is the sole source of funding and sponsor of this research.

## CRediT authorship contribution statement

**E.G. Tane:** Writing – review & editing, Writing – original draft, Investigation, Formal analysis, Data curation. **L. Martínez-Gómez:** Writing – review & editing, Writing – original draft, Investigation, Formal analysis, Data curation. **A. Amorós-Pérez:** Writing – review & editing, Writing – original draft, Investigation, Formal analysis, Data curation. **M.C. Román-Martínez:** Writing – review & editing, Writing – original draft, Visualization, Validation, Supervision, Methodology, Investigation, Formal analysis, Data curation, Conceptualization. **M.A. Lillo-Ródenas:** Writing – review & editing, Writing – original draft, Visualization, Validation, Supervision, Methodology, Investigation, Funding acquisition, Formal analysis, Data curation, Conceptualization.

## Declaration of competing interest

The authors declare the following financial interests/personal relationships which may be considered as potential competing interests:

The present study has been funded by Philip Morris Products, S.A.
