# Supplementary material for: A novel approach to the quantitative analysis of the particulate matter in conventional cigarette smoke and heated tobacco product aerosols
Source: Heliyon. 2024 Jul 25;10(15):e35028. doi: 10.1016/j.heliyon.2024.e35028 (PMC11336348; doi:10.1016/j.heliyon.2024.e35028)
Supplement: Multimedia component 1 [file mmc1.docx]

# SUPPLEMENTARY MATERIAL


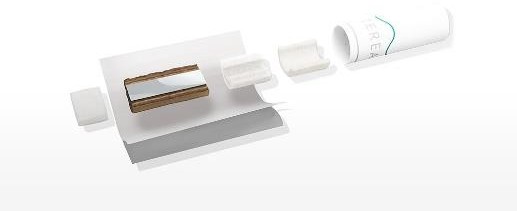


**(b)**


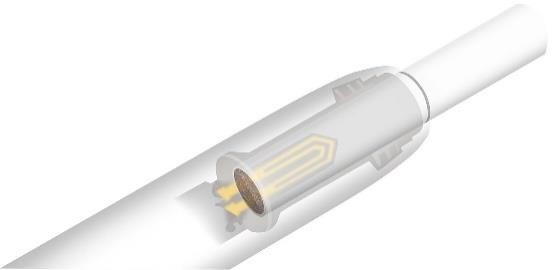


**(a)**

Figure SM1. Technology used in these different IQOS models: (a) blade system in IQOS 3 MULTI and IQOS 3 DUO, and (b) induction heating in IQOS ILUMA.


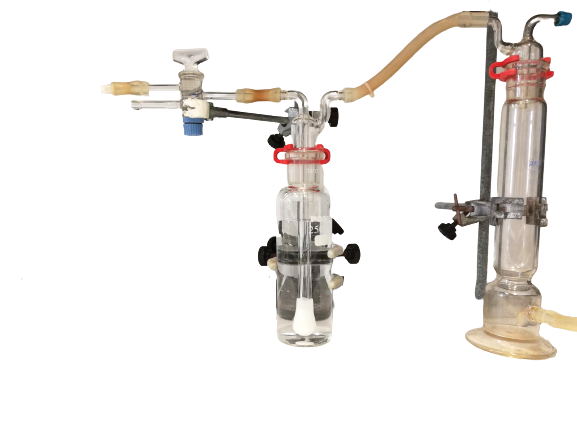


**Vacuum pump**

**Cigarette**

**IQOS**

Gas washing flask with water

Empty flask

Glass stopcock

Holey rubber piece

Extraction for GC analysis

*Figure SM2. Experimental setup to simulate cigarettes smoking or heatsticks use. Image redrawn from [14,16].*


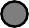

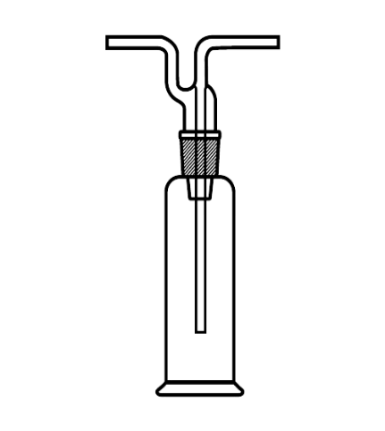

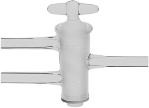

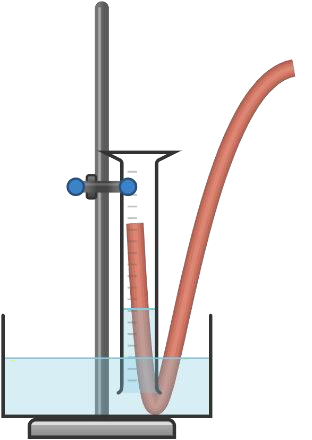

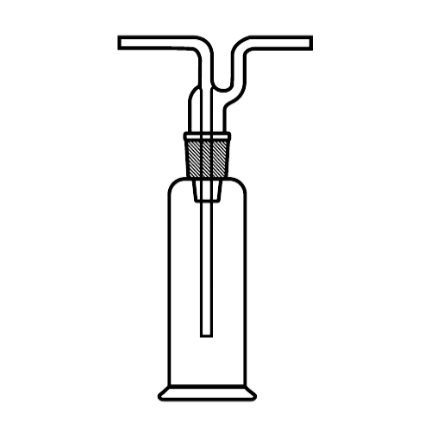

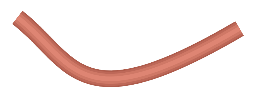

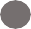

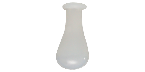


Vacuum pump

Figure SM3. Scheme of the flow rate determination process.


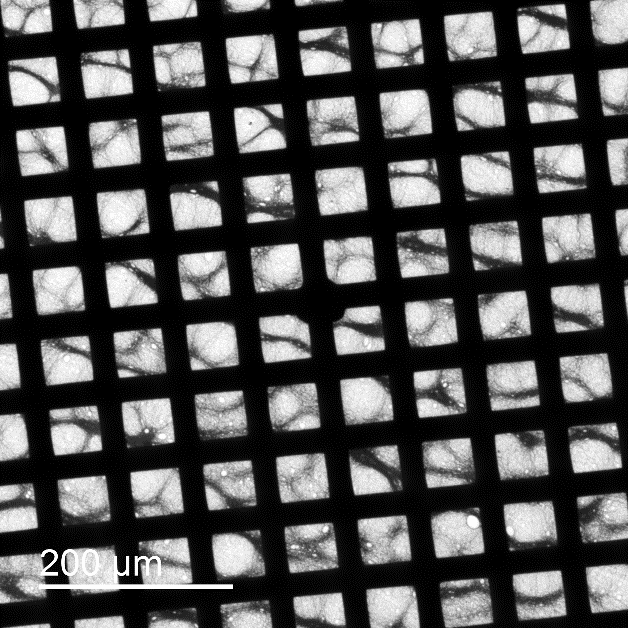


*Figure SM4. Overall image taken at 15x magnification for the water Blank A.*


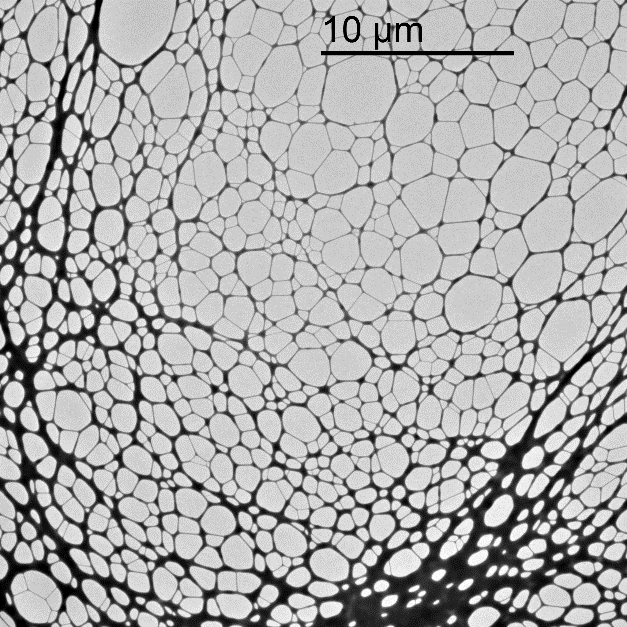


*Figure SM5. The centre of the image, taken at 300x magnification for the water Blank A.*


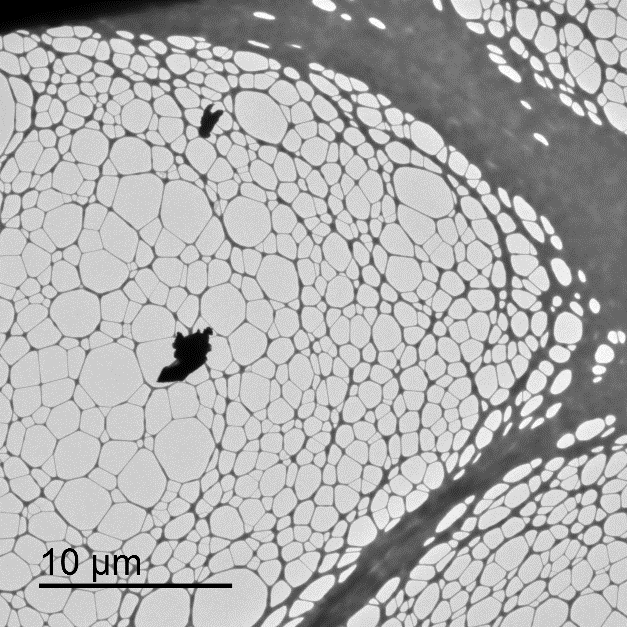


*Figure SM6. The image where most of the particulate matter are visible, taken at 300x magnification, for the water Blank A.*


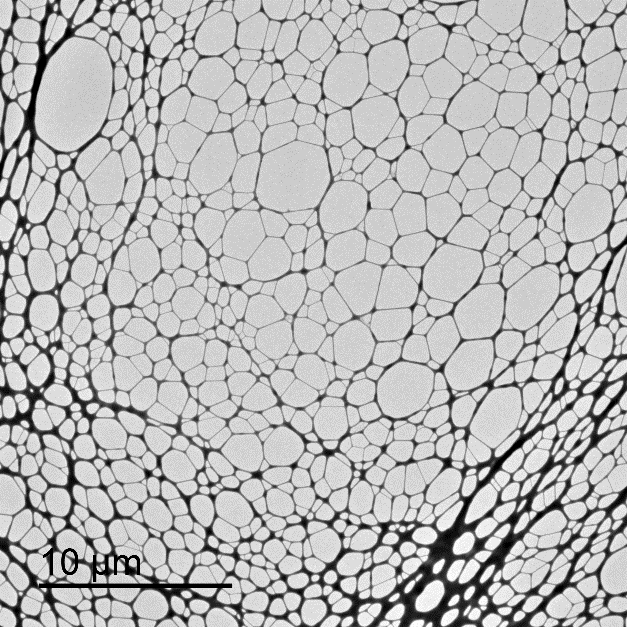


*Figure SM7. The image where very few or no particulate matter are visible, taken at 300x magnification, for the water Blank A.*


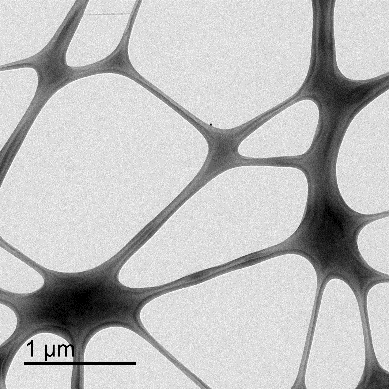

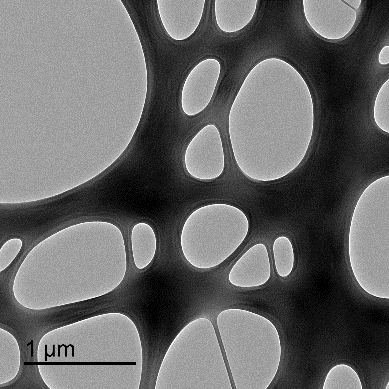

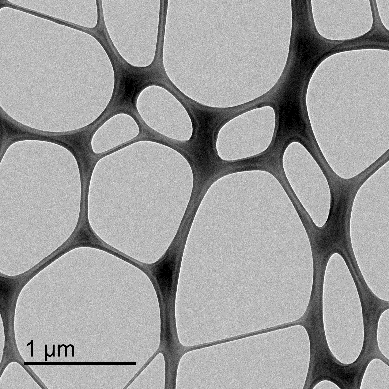

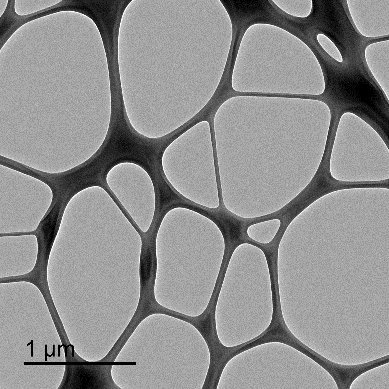


*Figure SM8. The images taken at coordinate A at 3000x magnification for the water Blank A.*


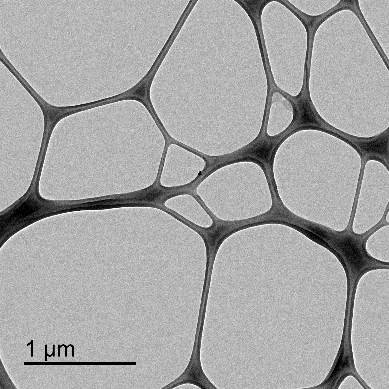

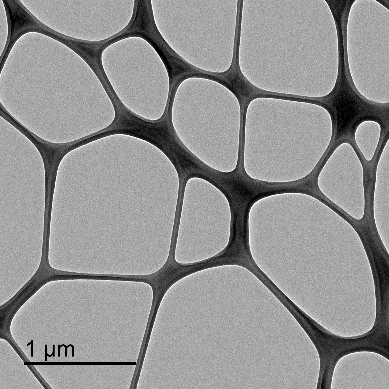

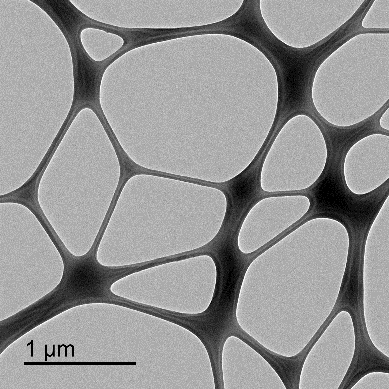

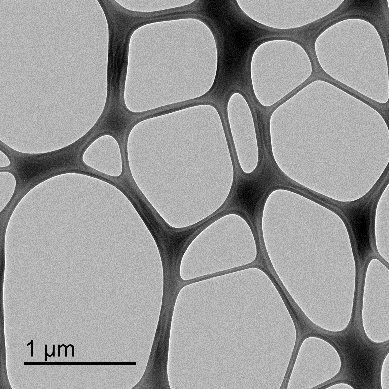


*Figure SM9. The images taken at coordinate B at 3000x magnification for the water Blank A.*


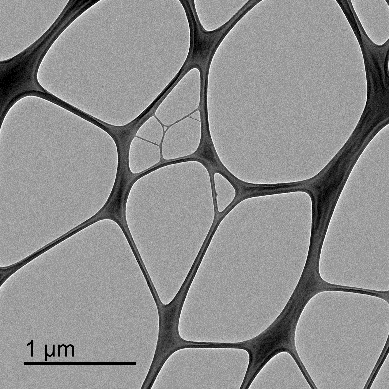

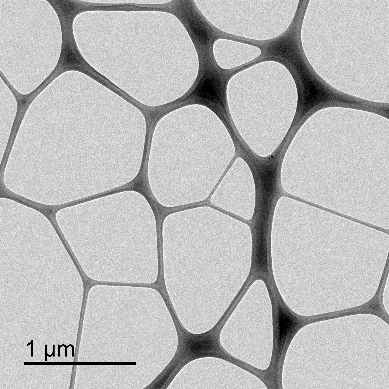

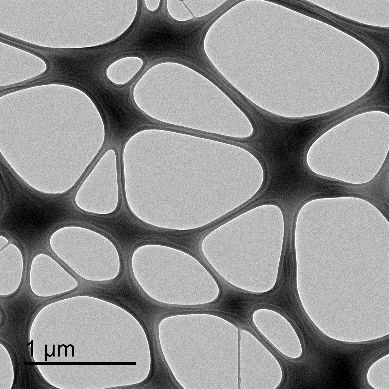

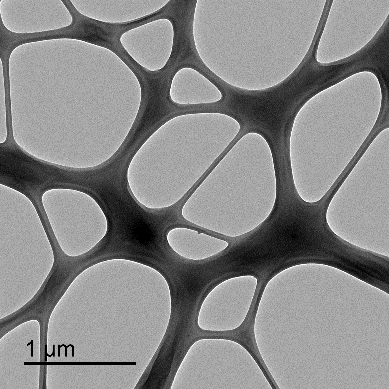


*Figure SM10. The images taken at coordinate C at 3000x magnification for the water Blank A.*


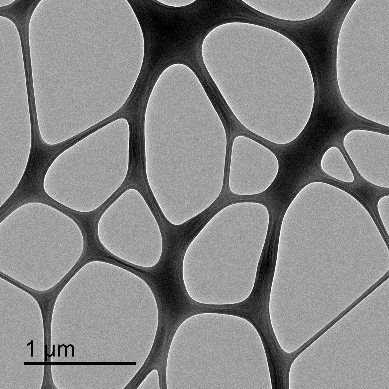

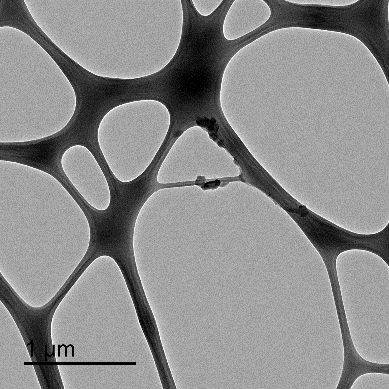

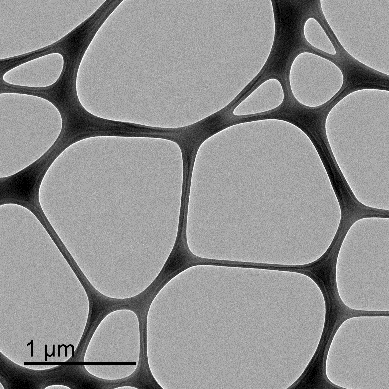

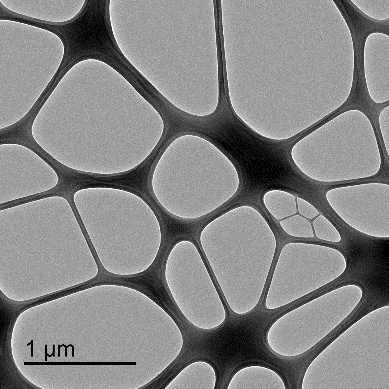


*Figure SM11. The images taken at coordinate D at 3000x magnification for the water Blank A.*


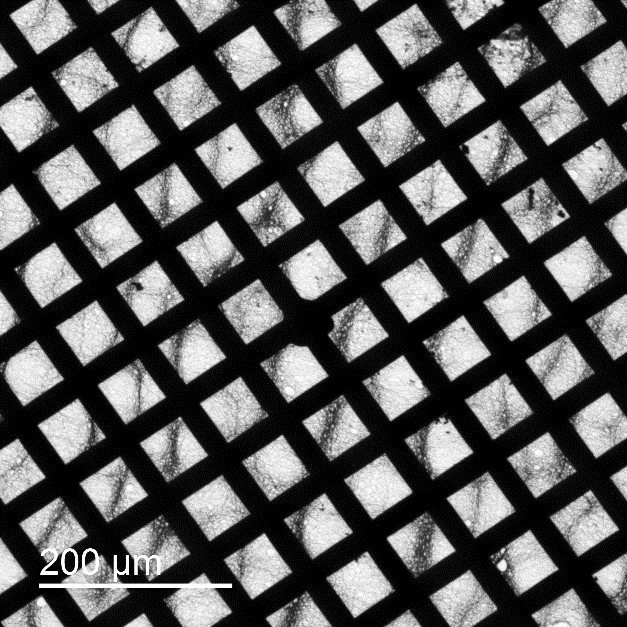


*Figure SM12. Overall image taken at 15x magnification for the CC sample (puffing experiment).*


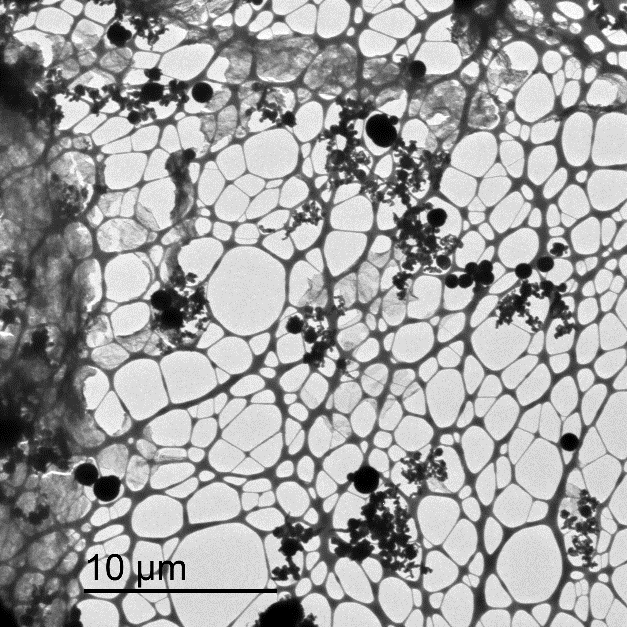


*Figure SM13. The centre of the image, taken at 300x magnification for the CC sample (puffing experiment).*


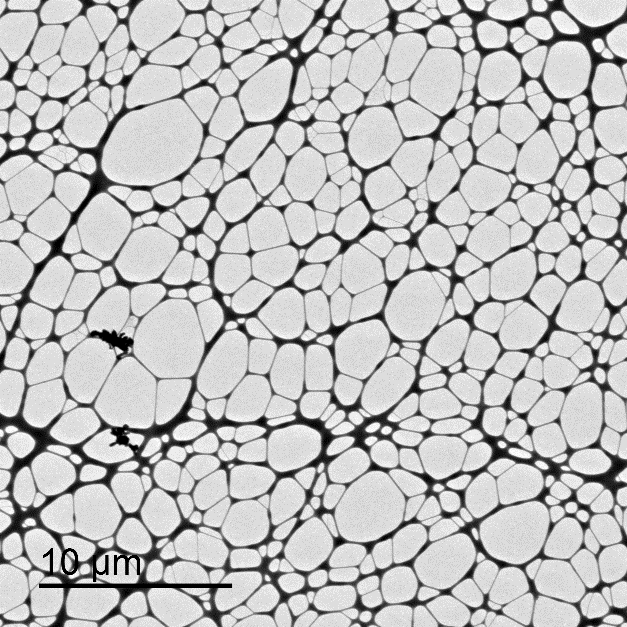


*Figure SM14. The image where most of the particulate matter are visible, taken at 300x magnification, for the CC sample (puffing experiment).*


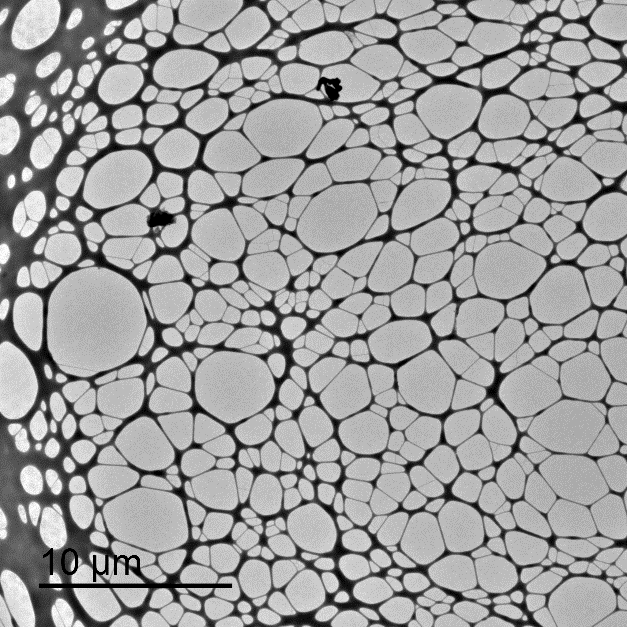


*Figure SM15. The image where very few or no particulate matter are visible, taken at 300x magnification, for the CC sample (puffing experiment).*


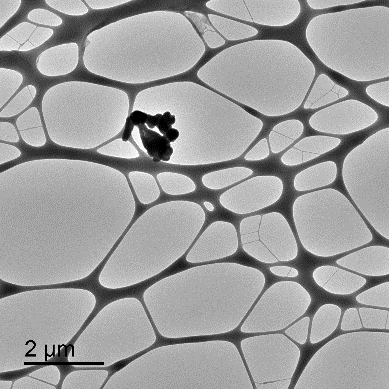

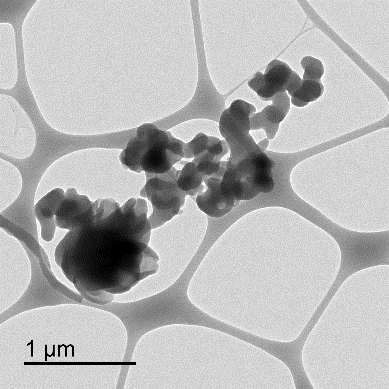

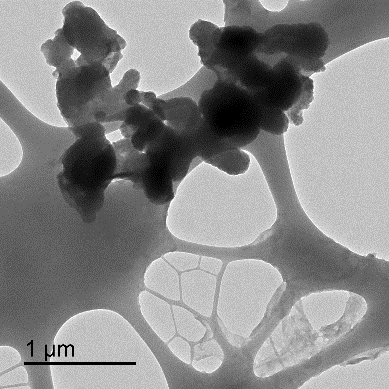

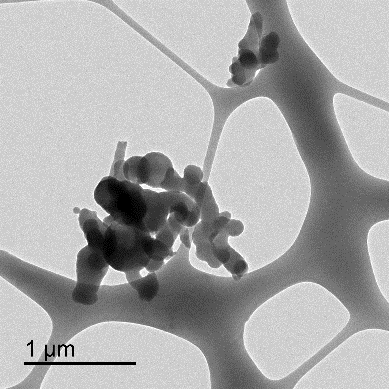


*Figure SM16. The sample taken at coordinate A at 3000x magnification for the CC sample (puffing experiment).*


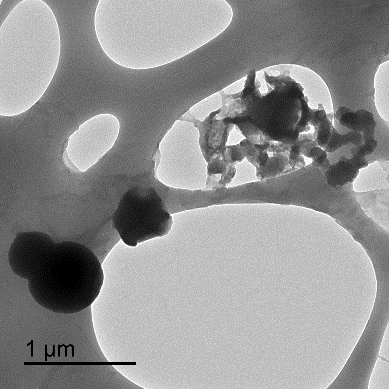

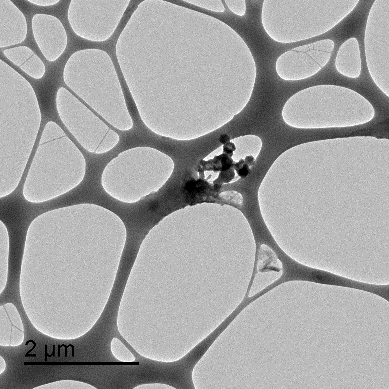

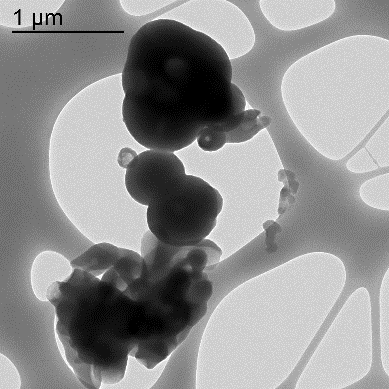

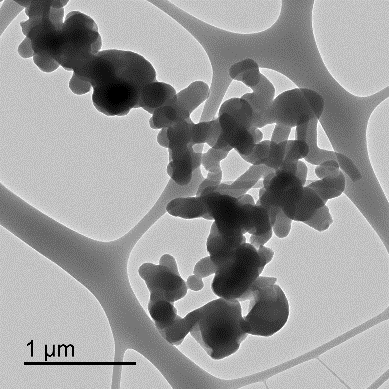


*Figure SM17. The sample taken at coordinate B at 3000x magnification for the CC sample (puffing experiment).*


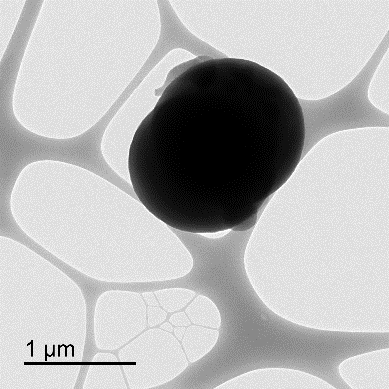

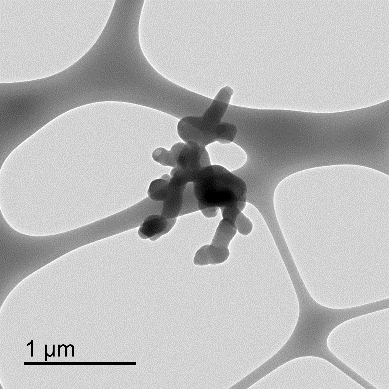

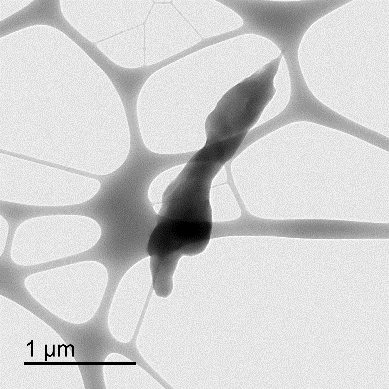

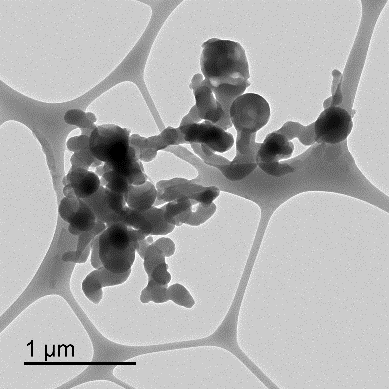


*Figure SM18. The images taken at coordinate C at 3000x magnification for the CC sample (puffing experiment).*


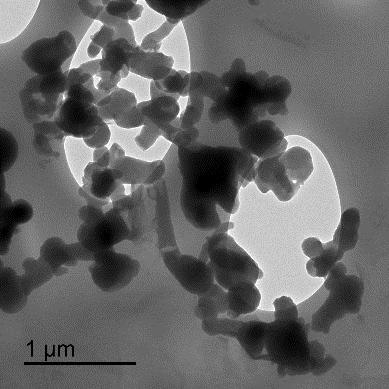

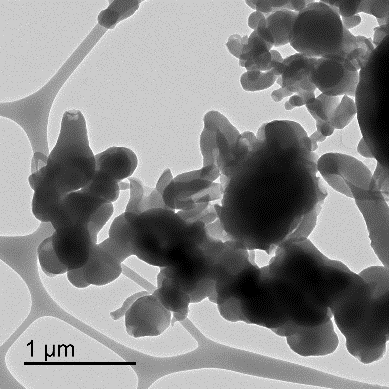

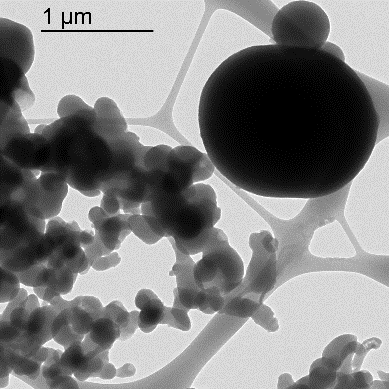

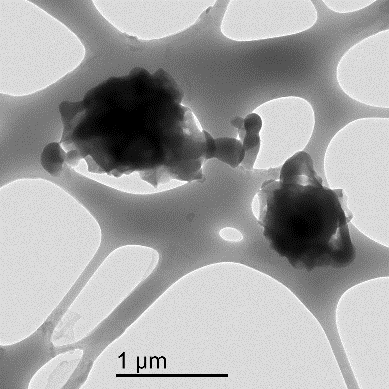


*Figure SM19. The images taken at coordinate D at 3000x magnification for the CC sample (puffing experiment).*


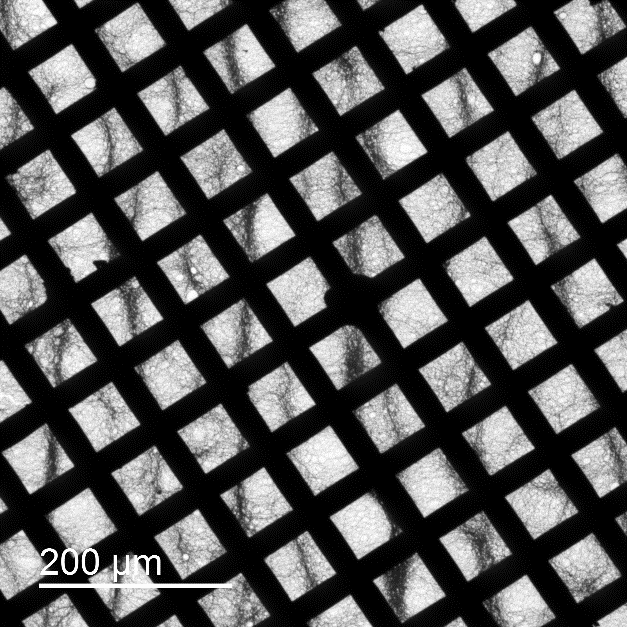


*Figure SM20. Overall image taken at 15x magnification for the HTP1 sample (puffing experiment).*


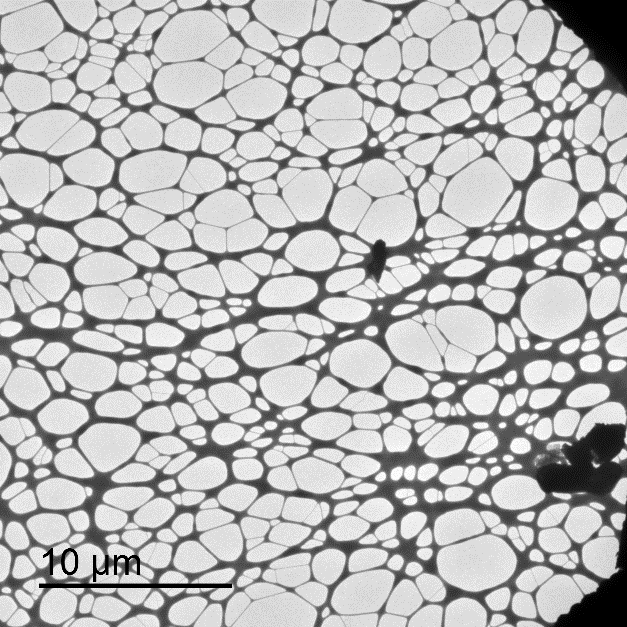


*Figure SM21. The centre of the image, taken at 300x magnification for the HTP1 sample (puffing experiment).*


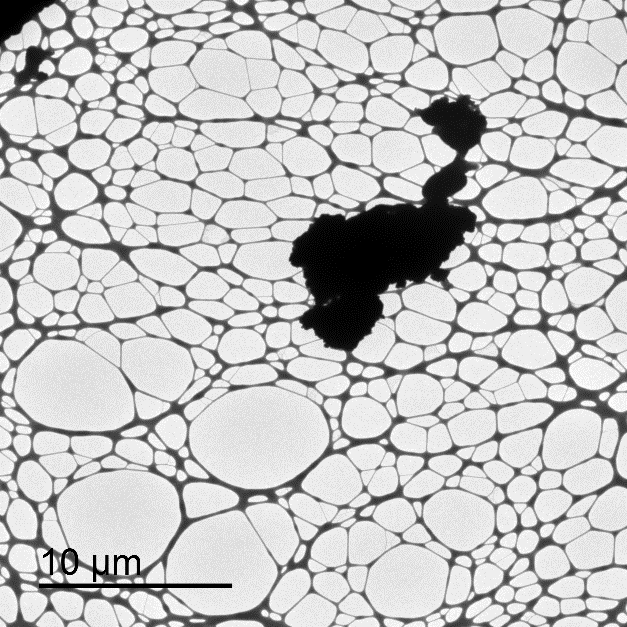


*Figure SM22. The image where most of the particulate matter are visible, taken at 300x magnification, for the HTP1 sample (puffing experiment).*


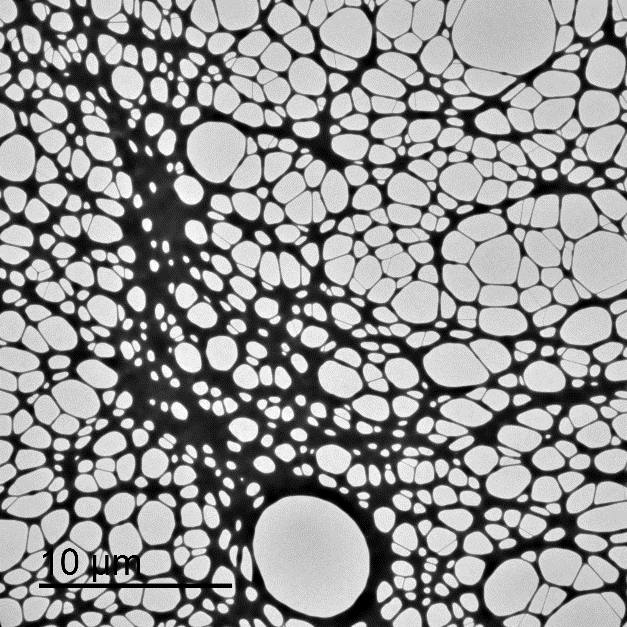


*Figure SM23 The image where very few or no particulate matter are visible, taken at 300x magnification, for the HTP1 sample (puffing experiment).*


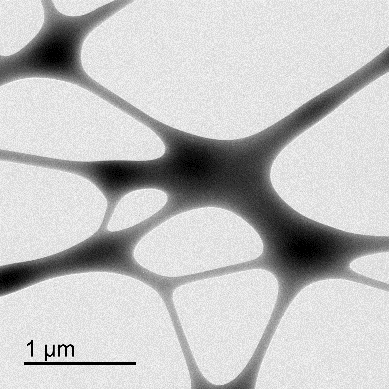

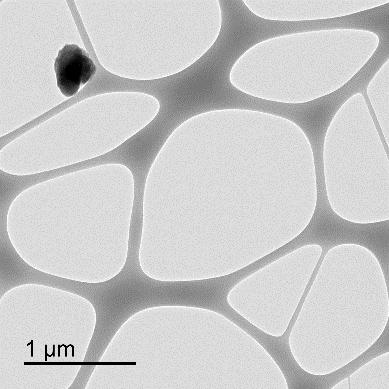

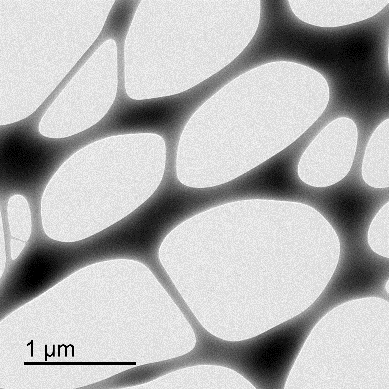

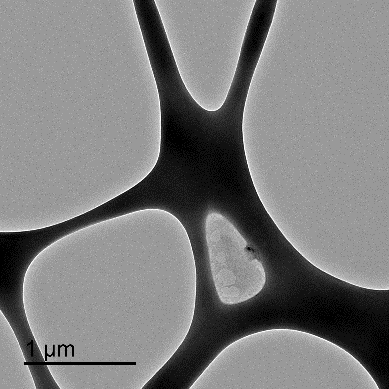


*Figure SM24. The images taken at coordinate A at 3000x magnification for the HTP1 sample (puffing experiment).*


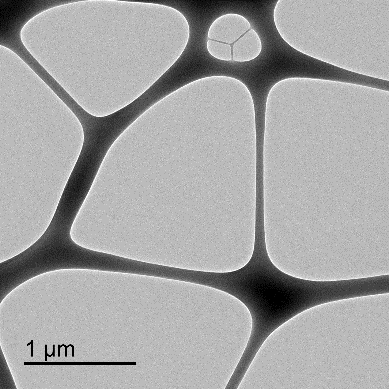

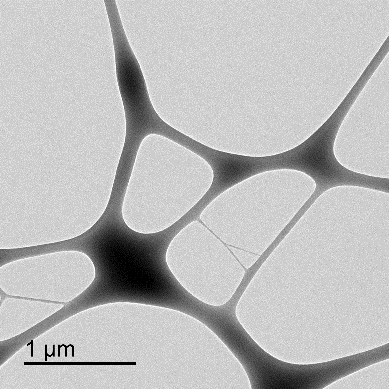

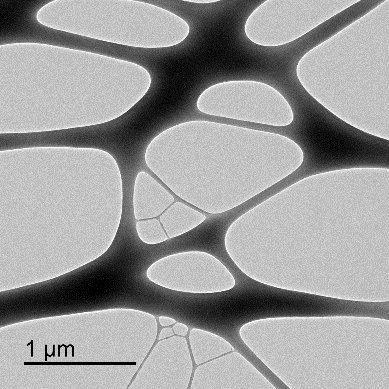

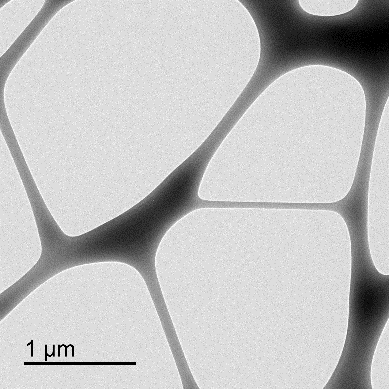


*Figure SM26. The images taken at coordinate C at 3000x magnification for the HTP1 sample (puffing experiment).*


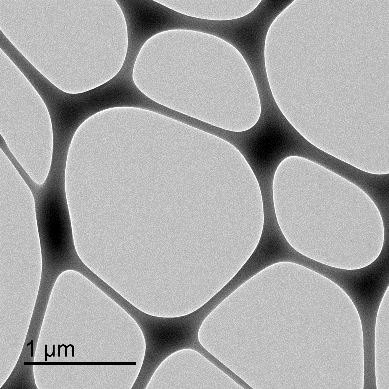

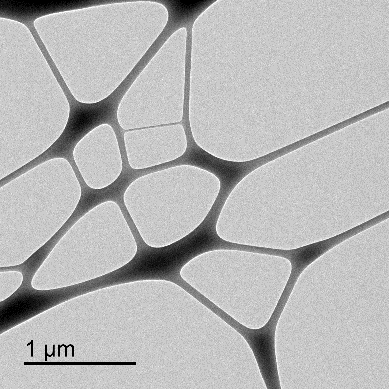

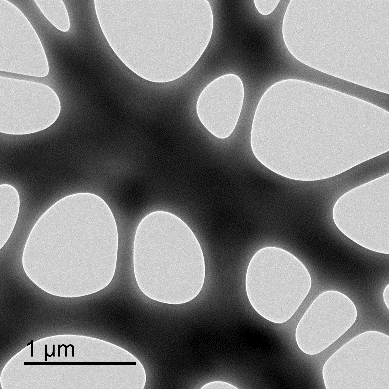

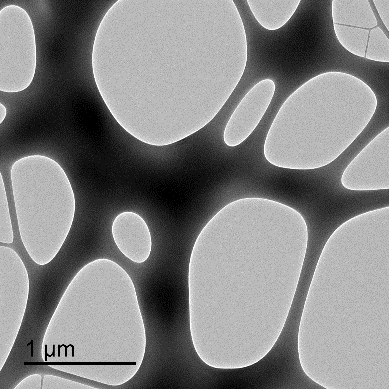


*Figure SM25. The images taken at coordinate B at 3000x magnification for the HTP1 sample (puffing experiment).*


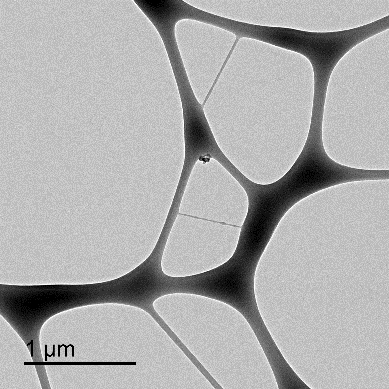

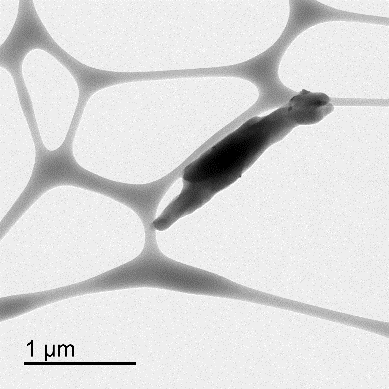

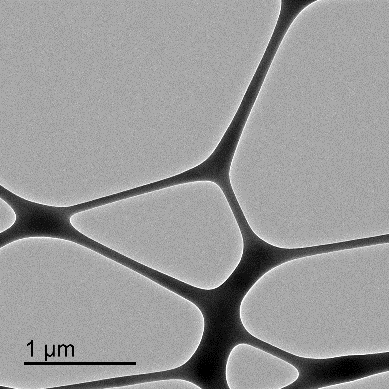

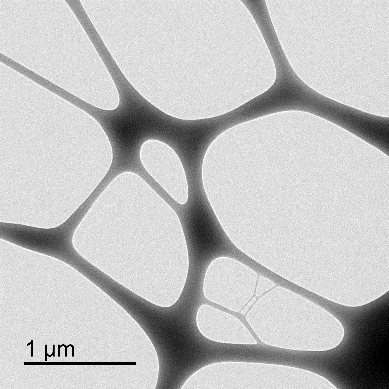


*Figure SM27. The images taken at coordinate D at 3000x magnification for the HTP1 sample (puffing experiment).*


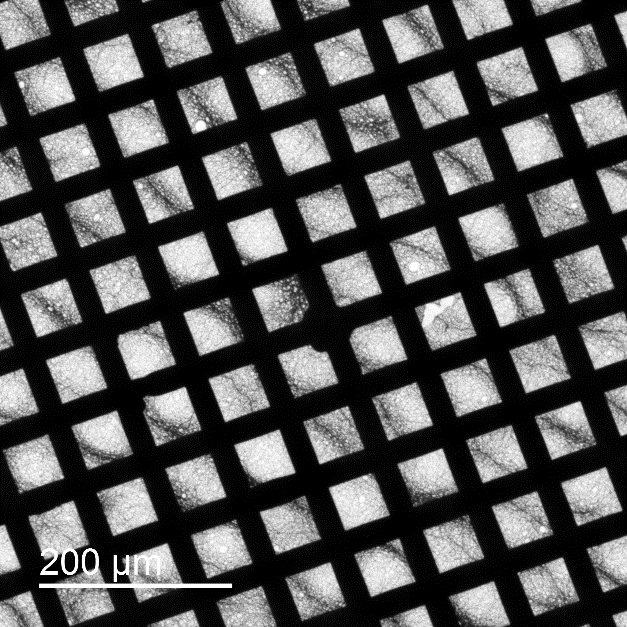


*Figure SM28. Overall image taken at 15x magnification for the HTP2 sample (puffing experiment).*


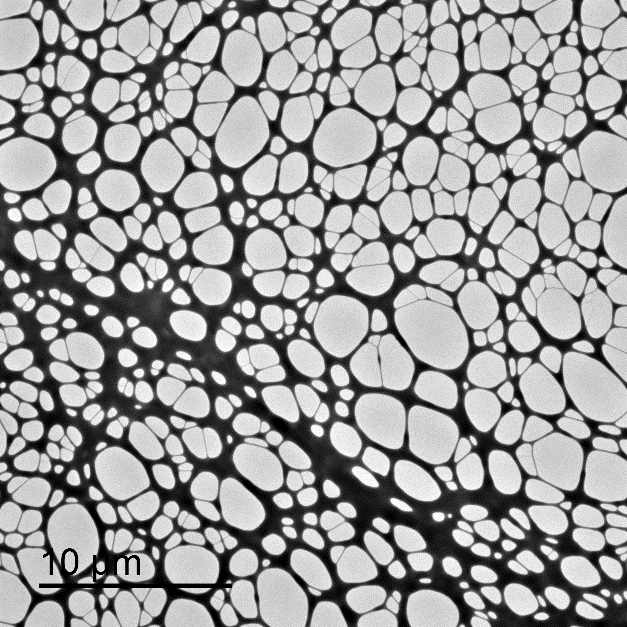


*Figure SM29. The centre of the image, taken at 300x magnification for the HTP2 sample (puffing experiment).*


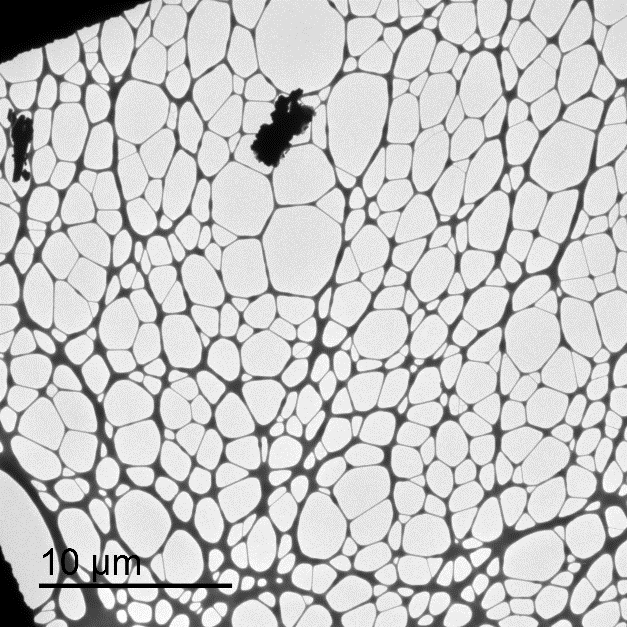


*Figure SM30. The image where most of the particulate matter are visible, taken at 300x magnification, for the HTP2 sample (puffing experiment).*


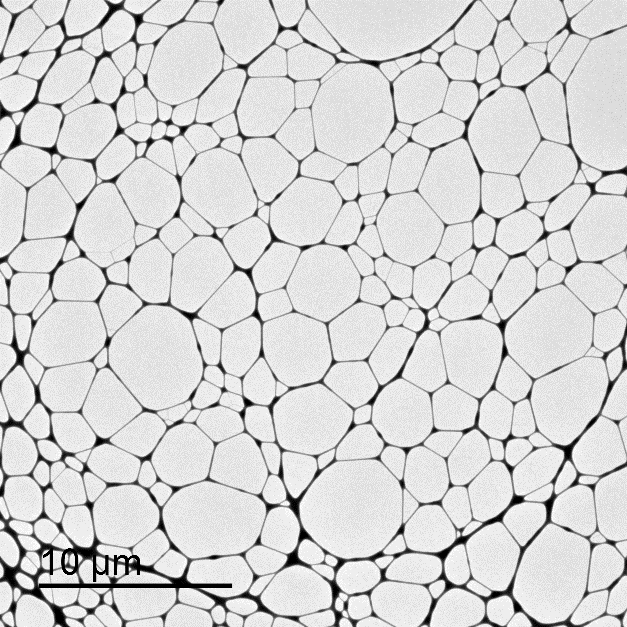


*Figure SM31. The image where very few or no particulate matter are visible, taken at 300x magnification, for the HTP2 sample (puffing experiment).*


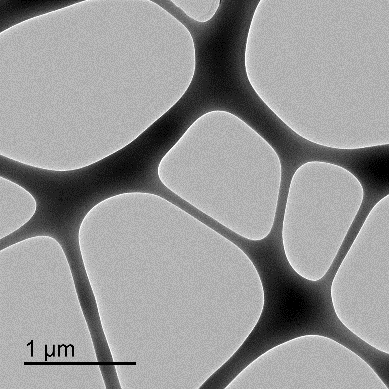

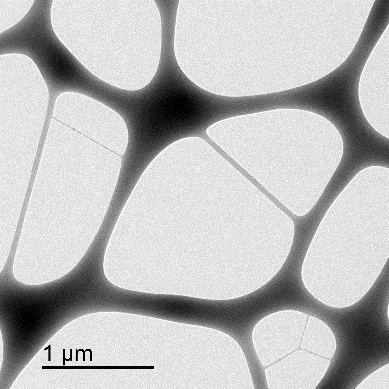

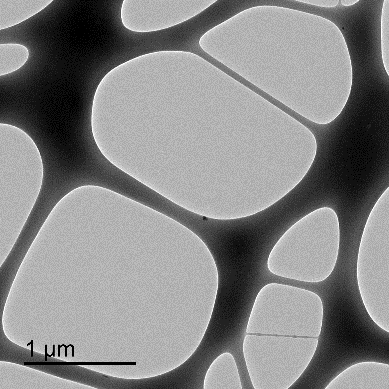

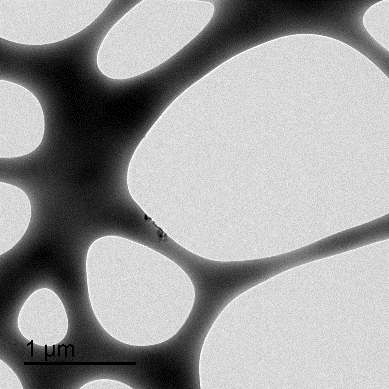


*Figure SM32. The images taken at coordinate A at 3000x magnification for the HTP2 sample (puffing experiment).*


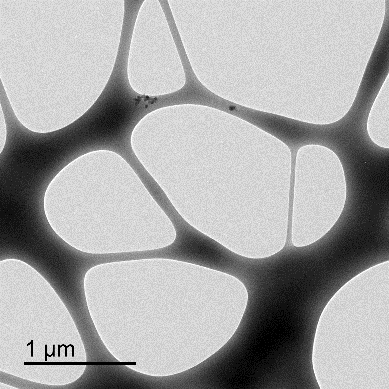

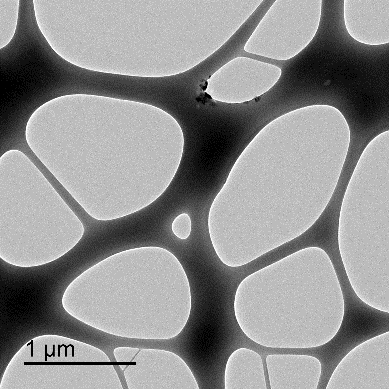

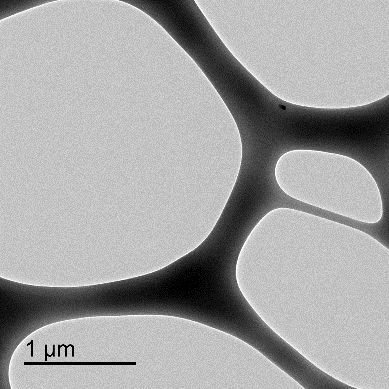

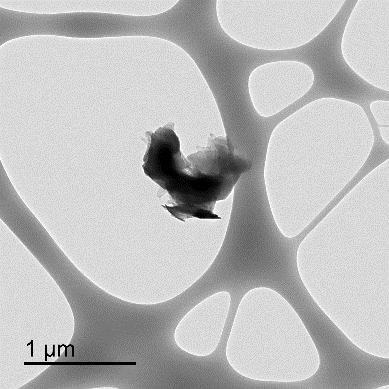


*Figure SM33. The images taken at coordinate B at 3000x magnification for the HTP2 sample (puffing experiment).*


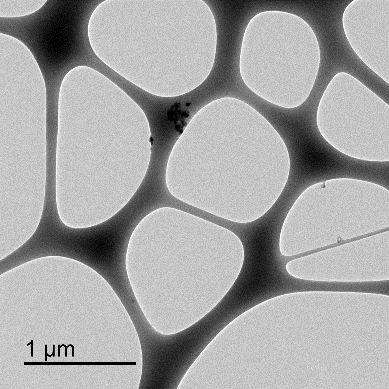

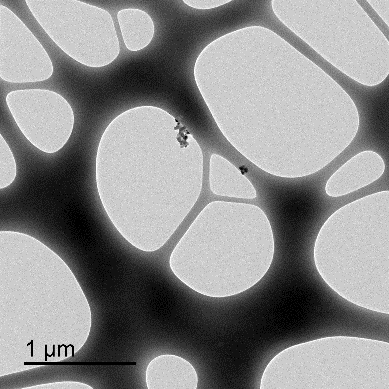

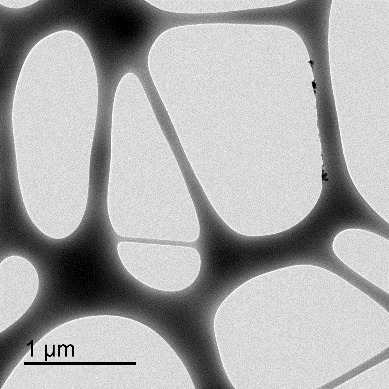

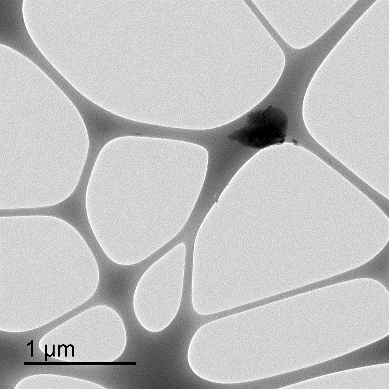


*Figure SM34. The images taken at coordinate C at 3000x magnification for the HTP2 sample (puffing experiment).*


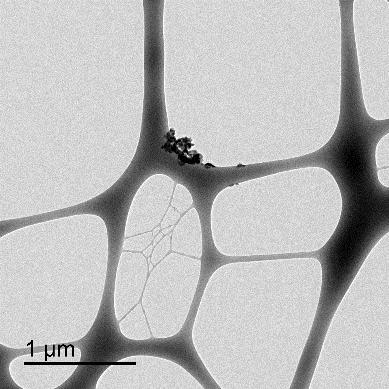

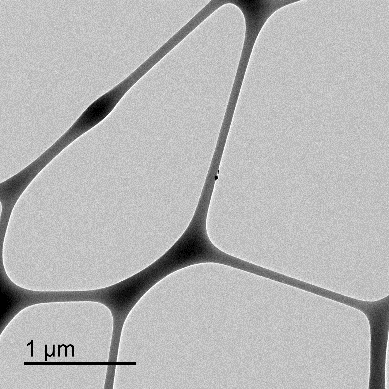

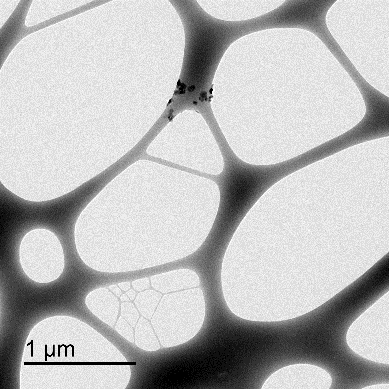

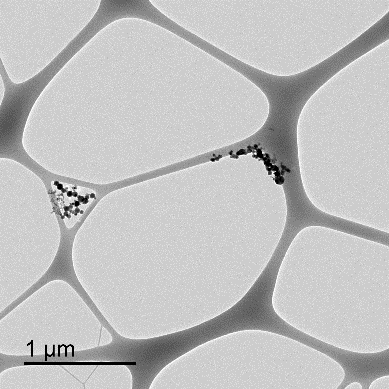


*Figure SM35. The images taken at coordinate D at 3000x magnification for the HTP2 sample (puffing experiment).*


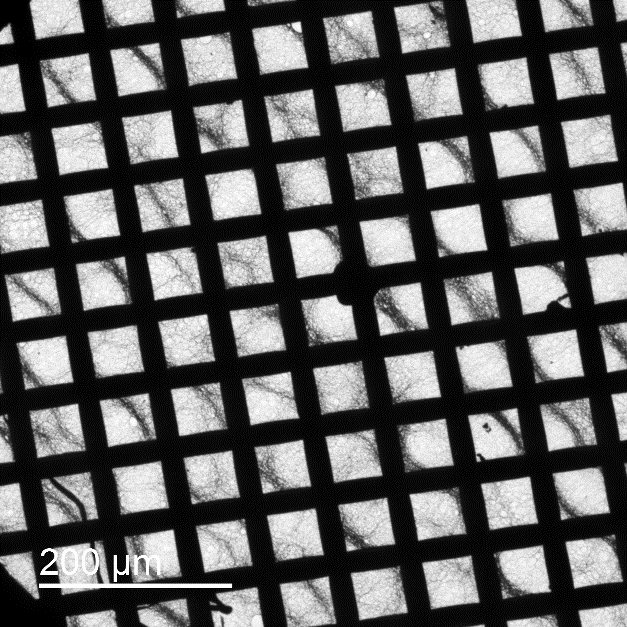


*Figure SM36. Overall image taken at 15x magnification for the HTP3 sample (puffing experiment).*


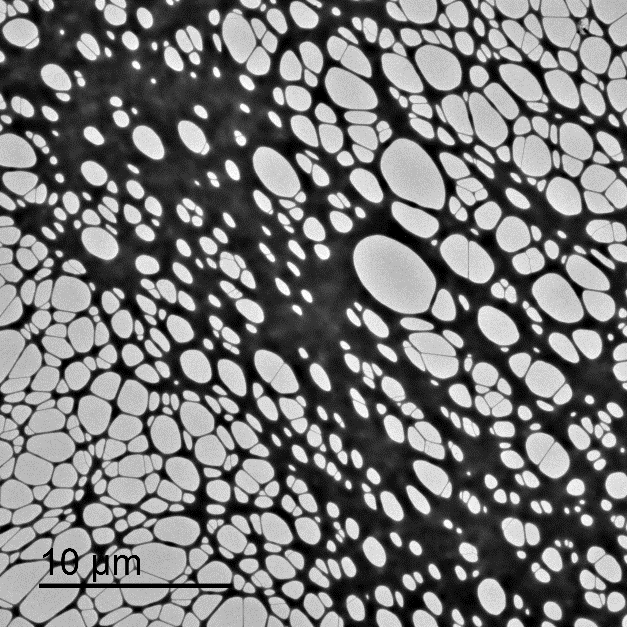


*Figure SM37. The centre of the image, taken at 300x magnification for the HTP3 sample (puffing experiment).*


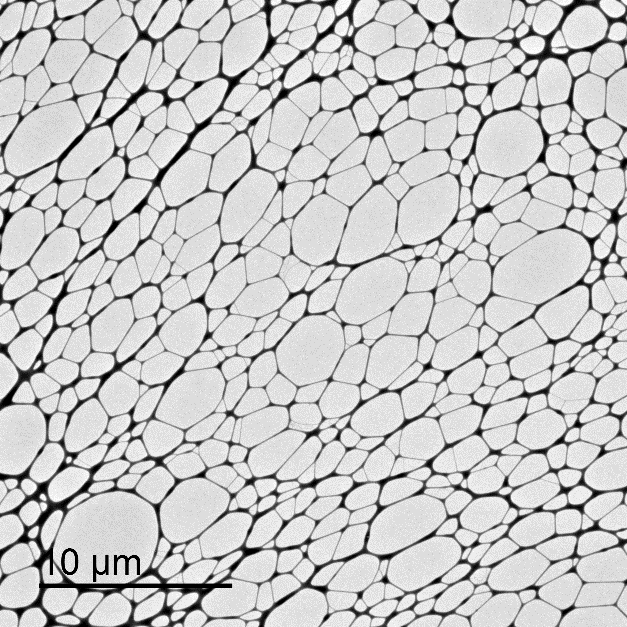


*Figure SM38. The image where most of the particulate matter are visible, taken at 300x magnification, for the HTP3 sample (puffing experiment).*


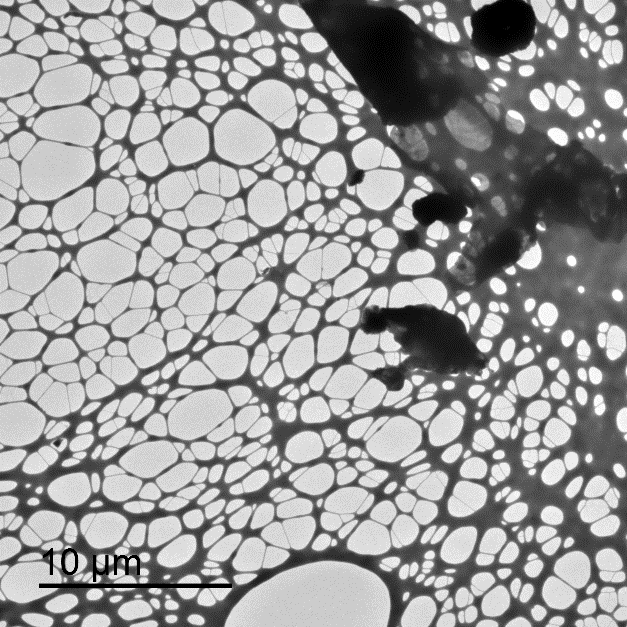


*Figure SM39. The image where very few or no particulate matter are visible, taken at 300x magnification, for the HTP3 sample (puffing experiment).*


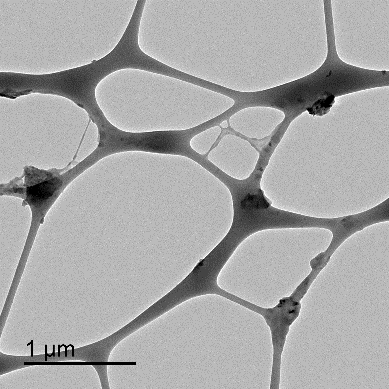

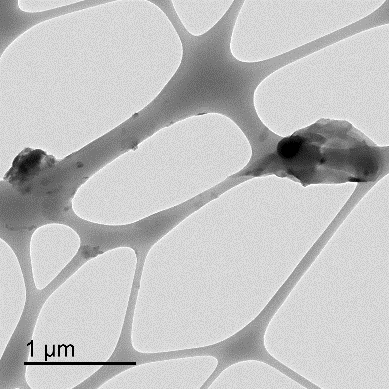

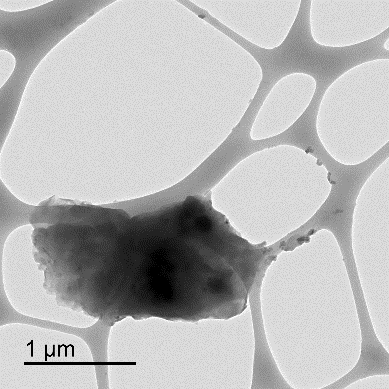

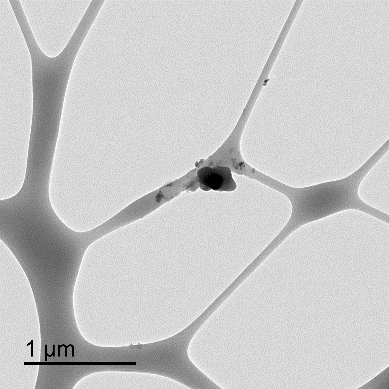


*Figure SM40. The images taken at coordinate A at 3000x magnification for the HP3 sample (puffing experiment).*


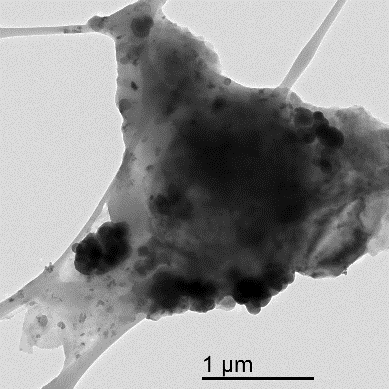


*Figure SM41. The images taken at coordinate B at 3000x magnification for HTP3 sample (puffing experiment).*

*Figure SM42. The images taken at coordinate C at 3000x magnification for the HTP3 sample (puffing experiment).*

*Figure SM43. The images taken at coordinate D at 3000x magnification for the HTP3 sample (puffing experiment).*
